# Thermosensitive collagen/fibrinogen gels loaded with decorin suppress lesion site cavitation and promote functional recovery after spinal cord injury

**DOI:** 10.1038/s41598-021-97604-w

**Published:** 2021-09-13

**Authors:** Jacob Matthews, Sarina Surey, Liam M. Grover, Ann Logan, Zubair Ahmed

**Affiliations:** 1 Neuroscience and Ophthalmology, Institute of Inflammation and Ageing, University of Birmingham, Edgbaston, Birmingham, B15 2TT UK; 2 School of Chemical Engineering, University of Birmingham, Edgbaston, Birmingham, B15 2TT UK; 3 Warwick Medical School, Biomedical Sciences, University of Warwick, Coventry, CV4 7AL UK; 4 Centre for Trauma Sciences Research, University of Birmingham, Edgbaston, Birmingham, B15 2TT UK

**Keywords:** Cellular neuroscience, Molecular neuroscience, Neurotrophic factors, Regeneration and repair in the nervous system

## Abstract

The treatment of spinal cord injury (SCI) is a complex challenge in regenerative medicine, complicated by the low intrinsic capacity of CNS neurons to regenerate their axons and the heterogeneity in size, shape and extent of human injuries. For example, some contusion injuries do not compromise the dura mater and in such cases implantation of preformed scaffolds or drug delivery systems may cause further damage. Injectable in situ thermosensitive scaffolds are therefore a less invasive alternative. In this study, we report the development of a novel, flowable, thermosensitive, injectable drug delivery system comprising bovine collagen (BC) and fibrinogen (FB) that forms a solid BC/FB gel (Gel) immediately upon exposure to physiological conditions and can be used to deliver reparative drugs, such as the naturally occurring anti-inflammatory, anti-scarring agent Decorin, into adult rat spinal cord lesion sites. In dorsal column lesions of adult rats treated with the Gel + Decorin, cavitation was completely suppressed and instead lesion sites became filled with injury-responsive cells and extracellular matrix materials, including collagen and laminin. Decorin increased the intrinsic potential of dorsal root ganglion neurons (DRGN) by increasing their expression of regeneration associated genes (RAGs), enhanced local axon regeneration/sprouting, as evidenced both histologically and by improved electrophysiological, locomotor and sensory function recovery. These results suggest that this drug formulated, injectable hydrogel has the potential to be further studied and translated into the clinic.

## Introduction

Spinal cord injury (SCI) is a leading cause of morbidity in the western world and currently there is little that can be done to improve functional recovery due to a lack of successful translational therapies^1,2^. The majority of SCI are caused by blunt trauma leading to an initial loss of function below the injury site, followed by secondary degeneration of the compromised neural tissue. Scar tissue quickly forms within the lesion site, constructing a physical and molecular barrier to regenerating axons. During the consolidation phase, fluid filled cysts develop within the scar tissue and surrounding area. These cysts swell into enlarged cavities that secondarily crush any surviving axons in the vicinity and cause an additional loss of distal function. If a treatment can be developed that can limit the scar and cavity formation this will almost certainly have beneficial effects for SCI patients by reducing the loss of function post-SCI and by increasing the potential for disinhibited spinal cord axon regeneration.

Scaffolds comprising growth permissive molecules provide a three-dimensional (3-D) environment to regenerating axons and also provide mechanical support for repair cells undergoing cell adhesion, migration and differentiation. Scaffolds composed of hydrogels are the most popular but nanofibers are being increasingly used to provide mechanical support and to deliver cells, drugs and growth factors^3^. Hydrogels are 3-D porous structures that have a high water content and many injectable hydrogels have been developed and tested in SCI scenarios^4,5^. Other scaffolds that are being developed and tested include both synthetic (e.g. poly(lactide) (PLA), poly(glycalide) (PGA)) and natural polymers (e.g. chitosan, agarose, alginate, collagen and fibrin)^6–9^. The bioactivities of scaffolds have also been enhanced by modification with, for example, cell adhesion peptides and pro-regenerative extracellular matrix (ECM) proteins such as fibronectin and laminin^9–14^.

Injectable hydrogels have drawn considerable interest in the last few years, especially for SCI where the dura mater is not compromised. In such cases in situ crosslinking hydrogels are particularly advantageous since they can fill the cavity with little further damage to the spinal cord architecture, thus eliminating problems associated with the excision of tissue^15^. Injectable scaffolds have been made from collagen, fibrin and fibrin + fibronectin and have been tested in a variety of SCI models^11,16–18^. Often, injectable scaffolds contain axon growth promoting drugs, including epidermal growth factor (EGF), fibroblast growth factor-2 (FGF-2), Chondroitinase ABC (ChABC), neurotrophin-3 (NT-3) and brain-derived neurotrophic factor (BDNF)^19–25^. Injectable in situ thermosensitive scaffolds are minimally invasive and hold some advantages, since these synthetic or natural polymers undergo a rapid liquid to gel transformation post-injection, conform to the individual’s lesion site and integrate well with the host tissue.

The release of the inflammatory cytokine transforming growth factor β1/2 (TGFβ1/2) into wounds from platelets and injury responsive cells creates a pro-inflammatory environment, activating gliosis, fibrotic scarring and neovascularisation, with levels rising rapidly after SCI^26,27^. Some elements of the nascent scar, including chondroitin sulphate proteoglycans (CSPG), actively inhibit axon growth by signalling axon growth cone collapse. Experimental administration of exogenous TGFβ1 increases ECM deposition in CNS lesions whilst antibodies to TGFβ1/2 or small molecule inhibitors suppress CNS scarring and inflammation^28,29^. Decorin is a naturally occurring anti-inflammatory molecule that is an antagonist of TGFβ1/2 and modulates diverse actions in the CNS, including suppressing scarring, inhibiting fibrogenesis, stimulating the secretion of plasminogen/plasmin to promote fibrolytic degradation of ECM and promoting axon regeneration^30–35^. We have shown previously that surgical implantation of freeze-dried collagen matrices injected with Decorin once implanated into dorsal column (DC) lesions either acutely or chronically attenuated, inflammation, gliosis, inhibitory scarring, spinal cord cavity size and promotes DC axon regeneration^36^. However, surgical implantation of a scaffold is not very translational and hence there is a drive towards creating thermosensitive hydrogels, that can be stored easily and be incorporated with therapeutic agents before injection into an SCI site^37^. These therapeutic scaffolds would then fill the cystic cavity and support axonal growth or cell differentiation^37^.

In the present study, we sought to determine the utility of an injectable Decorin-loaded thermosensitive bovine collagen/fibrinogen (BC/FB) hydrogel to promote remodelling of spinal cord tissues and functional recovery after DC lesion in adult rats.

## Results

### Characterisation of the bovine collagen/fibrinogen (BC/FB) gel

**Figure 1:**
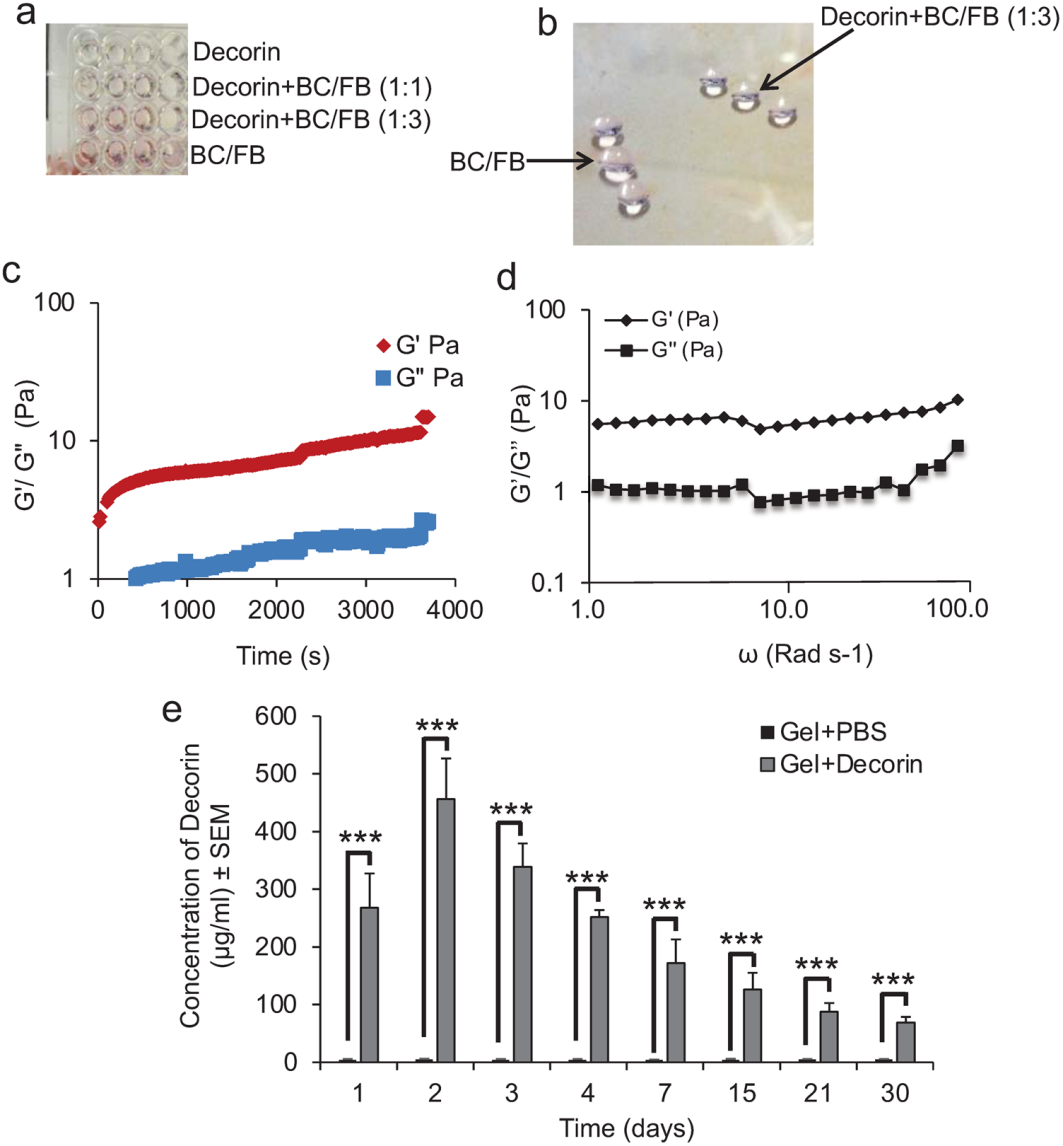
In vitro properties of injectable Gels. (**a**) BC/FB formed Gels when incubated at 37 °C in 96-well plates and could still polymerise into a Gel, enough to be scooped out (**b**) when 25% of its volume was Decorin (in PBS) (i.e. 1:3 Decorin:BC/FB) but not when 50% of its total volume was Decorin (i.e. 1:1 Decorin: BC/FB). (**c**) Frequency time-sweep measured the stability of the Gel over time. (**d**) The mechanical properties of the Gel were assessed by measuring the storage modulus (G’) and loss modulus (G’’) across a range of angular frequencies, ω. (**e**) Decorin or PBS was preloaded into the BC/FB Gels and was released when placed into 1 ml of PBS at increasing concentrations/day to a maximum at 2 days, declining gradually over the next 30 days. However, significant amounts of Decorin were still being released from the Gel at 15 days. ***P < 0.0001, one-way ANOVA with Dunnett’s post hoc test, Gel + Decorin *vs* Gel + PBS. *n* = 4 Gels/group, 3 independent repeats, total *n* = 12 Gels/group. No Decorin was detected in Gel + PBS-treated groups.

We first investigated the gelling properties of the BC/FB gel (termed Gel from herein) and determined that the Gel formed within 1 min of incubation at 37 °C (Fig. 1a). At this point it was possible to scoop this out of 96-well plates as an intact Gel (Fig. 1b). It was also possible to scoop the Gel out of the 96-well plates when the Gel was mixed with 1 part water to 3 parts BC/FB (Fig. 1b). However, the addition of 1 part water to 1 part BC/FB did not allow a gel to form and at this point it was not possible to scoop the Gel out of 96-well plates. This result meant that we could add 1-part Decorin solution to 3 parts BC/FB and still retain the gelling properties.

We then determined the characteristics of the thermosensitive Gel by performing rheology. Oscillatory time sweeps demonstrated that the gel became a stable structure after 800 s, beyond which there were very small changes in elastic modulus, G’, and the plastic modulus, G’’, (Fig. 1c), whilst the mechanical properties (elastic and plastic modulus) of the gel increased gradually with angular frequency, ω, but the increases were reminiscent of a relatively weak gel (Fig. 1d).

### Release profile of Decorin from Gels in vitro

To determine the release profile of Decorin from the Gel in vitro, we determined the concentration of Decorin released into PBS over a 30-day period at 37 °C. No Decorin was detected by ELISA in Gel + PBS control groups, however, in Gel + Decorin groups, Decorin was released into solution at a rate of 268 ± 59 µg/ml/d after 1 d, peaking at 457 ± 40 µg/ml at 2 days (Fig. 1e). The rate of Decorin release reduced to 252 ± 13 µg/ml and 156 ± 71 µg/ml at 3 and 4 days, respectively (Fig. 1c). Decorin continued to be released from the Gel in significant amounts for up to 30 days (*P* < 0.0001, ANOVA) with 69 ± 10 µg/ml released into solution at 30 days (Fig. 1c). These results demonstrated that the Gels release Decorin in a sustained manner and pharmacologically significant amounts are released in vitro for up to 30 days.

### Gel + Decorin promotes DRGN survival and neurite outgrowth in vitro

**Figure 2:**
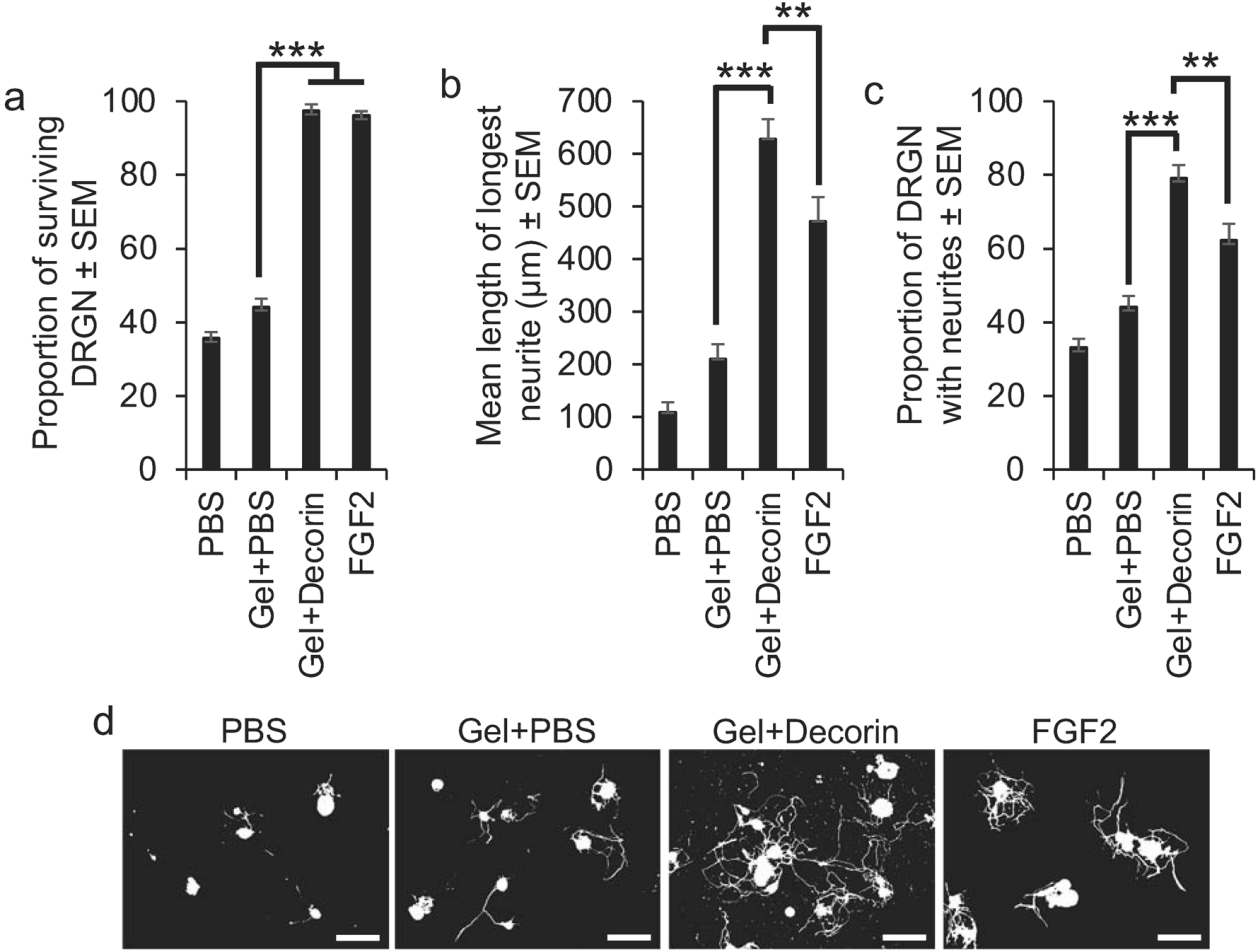
Gel + Decorin promotes DRGN survival and neurite outgrowth in vitro in a serum-withdrawal model. (**a**) Gel + Decorin increased the proportion of surviving DRGN, which was similar to that observed with the positive control, fibroblast growth factor-2 (FGF2). Gel + Decorin treatment also increased (**b**) the mean DRGN neurite length and (**c**) the proportion of DRGN with neurites. (**d**) Representative photomicrographs to show neurite outgrowth after PBS, Gel + PBS, Gel + Decorin and FGF2 treatment. Scale bars in (**d**) = 100 µm. **P < 0.001; ***P < 0.0001, one-way ANOVA with Dunnett’s post hoc test. *n* = 3 wells/condition, 3 independent repeats, total *n* = 9 wells/condition.

To determine if the released amount Decorin from the Gel is sufficient to promote DRGN survival and neurite outgrowth in a serum withdrawal model in vitro, Gels were incubated with cultured DRGN in serum-free DMEM, which is normally insufficient to promote DRGN survival and over 5 days there is > 60% DRGN death^38^. In PBS-treated cultures, only 40% of DRGN survived after 5 days (Fig. 2a). There was a slight improvement in DRGN survival in Gel + PBS-treated cultures but incubation with Gel + Decorin was able to promote near 100% survival for the full 5 days (*P* < 0.0001. ANOVA; Fig. 2a), with DRGN survival being similar to positive control FGF2-treated wells (Fig. 2a). Gel + Decorin also promoted significantly increased neurite outgrowth in terms of the mean longest neurite length (416 ± 39 µm vs 210 ± 27 µm; *P* < 0.0001, ANOVA; Fig. 2b,d) and the proportion of DRGN with neurites (65 ± 6 vs 42 ± 4%; *P* < 0.0001, ANOVA; Fig. 2c,d) compared to Gel + PBS-treated groups. The mean longest DRGN neurite length and the proportion of DRGN with neurites were also significantly greater in the Gel + Decorin groups compared to the positive FGF2 control groups (*P* < 0.001, ANOVA; Fig. 2b–d). These results demonstrated that the released amounts of Decorin from Gels in vitro were sufficient to promote significant DRGN survival and neurite outgrowth. DRGN survival was comparable to FGF2 but neurite outgrowth was significantly greater than that observed in FGF2-treated positive control cultures.

In separate experiments, we determined the neuroprotective effect of Decorin alone (i.e. without Gels) to DRGN, by incubating DRGN with different concentrations of Decorin, directly onto the cultures and incubated them for 5 days before assessment of DRGN survival and neurite outgrowth. We demonstrate that Decorin was neuroprotective in a dose-dependent manner, promoting maximal DRGN survival at 250 µg/ml, where nearly 100% of DRGN survived, comparable to the positive control FGF2 (*P* < 0.0001, ANOVA; Fig. S1a). Concentrations of Decorin above 750 µg/ml appeared to be toxic to DRGN, reducing the proportion of surviving DRGN (Fig. S1a). The optimal concentration of Decorin (i.e. 250 µg/ml) that promoted maximal DRGN survival also promoted maximum DRGN neurite outgrowth in terms of the mean longest neurite length (*P* < 0.0001, ANOVA; Fig. S1b) and the number of DRGN with neurites (Fig. S1c). Once again, DRGN survival was comparable but neurite outgrowth was significantly increased in Decorin-treated cultures compared to FGF2-treated cultures (*P* < 0.001. ANOVA; Fig. S1a–c). These results suggest that Decorin is DRGN neuroprotective and neuritogenic.

### Gel + Decorin treatment suppressed cavitation after DC crush injury in vivo

**Figure 3:**
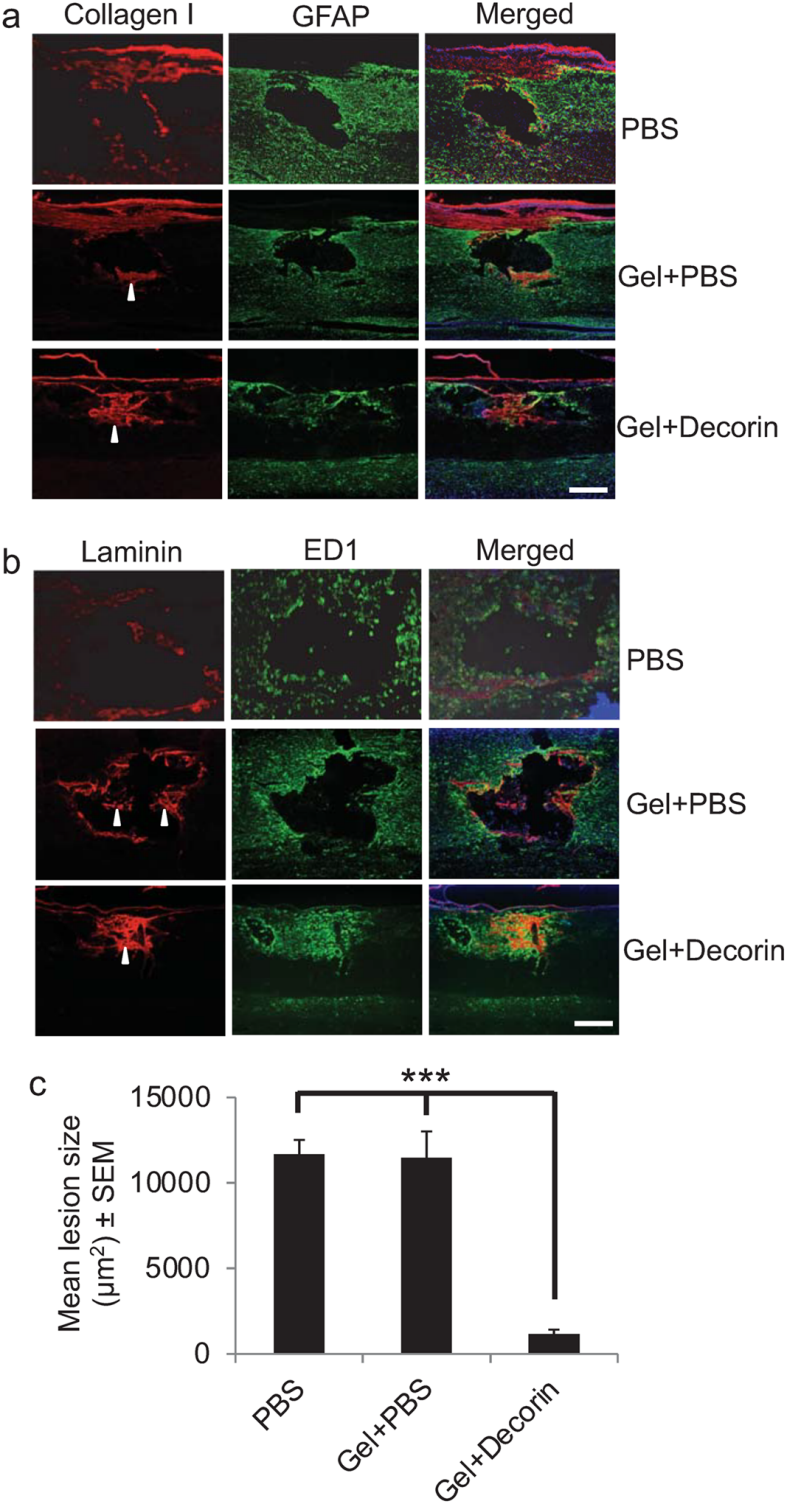
Gel + Decorin enhances deposition of ECM molecules within the DC wound core and reduces cavity size. (**a**) DC injury + PBS treatment normally generates a cavity in the lesion site but even with Gel + PBS small amounts of collagen I persisted (arrowhead) within the lesion site. However, in Gel + Decorin-treated lesion sites, both (**a**) collagen I (arrowhead) and (**b**) laminin (arrowhead) filled the lesion core. (**a**) GFAP and (**b**) ED1^+^ cells filled the lesion site after Gel + Decorin treatment but were restricted to the cavity edge in Gel + PBS injected rats. (**c**) Gel + Decorin injections significantly suppressed wound cavity size. ***P < 0.0001, one-way ANOVA with Dunnett’s post hoc test. Scale bars in (**a**) and (**b**) = 200 µm. *n* = 4 rats/group, 3 independent repeats, total *n* = 12 rats/group.

To determine if the Gel and Gel + Decorin was able to suppress SCI-induced cavitation in the rat, we injected Gel + PBS and Gel + Decorin into DC crush injury sites and performed immunohistochemistry at 6 weeks for a variety of markers including ECM markers, laminin and collagen I, and cellular markers such as ED1 and GFAP. In PBS-treated wounds, a significant cavity was observed, lined by GFAP^+^ astrocytes, with little residual immunostaining for collagen I and laminin within the cavity (Fig. 3a,b). In Gel + PBS-treated animals, a significant cavity was also observed, again lined by GFAP^+^ astrocytes, but with some immunostaining for collagen I and laminin within the cavity (Fig. 3a,b: arrowheads). However, in Gel + Decorin injected groups, collagen I and laminin immunoreactivity filled the lesion core with evidence of GFAP^+^ astrocyte invasion into the lesion site (Fig. 3a,b). Immunostaining for ED1 and GFAP in PBS and Gel + PBS-treated groups, laminin and ED1^+^ macrophage immunoreactivity was present at the edges of the cavity, whilst wound sites injected with Gel + Decorin were filled with laminin-rich material plus abundant ED1^+^ immunoreactivity throughout the lesion site (Fig. 3b). Quantification of the mean lesion size showed that injectable Gel + Decorin significantly suppressed DC cavity area by 92% compared with PBS and Gel + PBS (12,304 ± 4567 μm^2^ *vs* 1107 ± 266 μm^2^; *P* < 0.0001, ANOVA; Fig. 3c). These results demonstrate that injections of Gel + Decorin into DC crush sites suppressed cavity formation, probably by protecting the ECM (including collagen I and laminin) deposited in the lesion site, thereby facilitating migration of repair cells (including glia and macrophages).

### Gel + Decorin injections stimulated local MMP activation and tPA secretion in vivo

**Figure 4:**
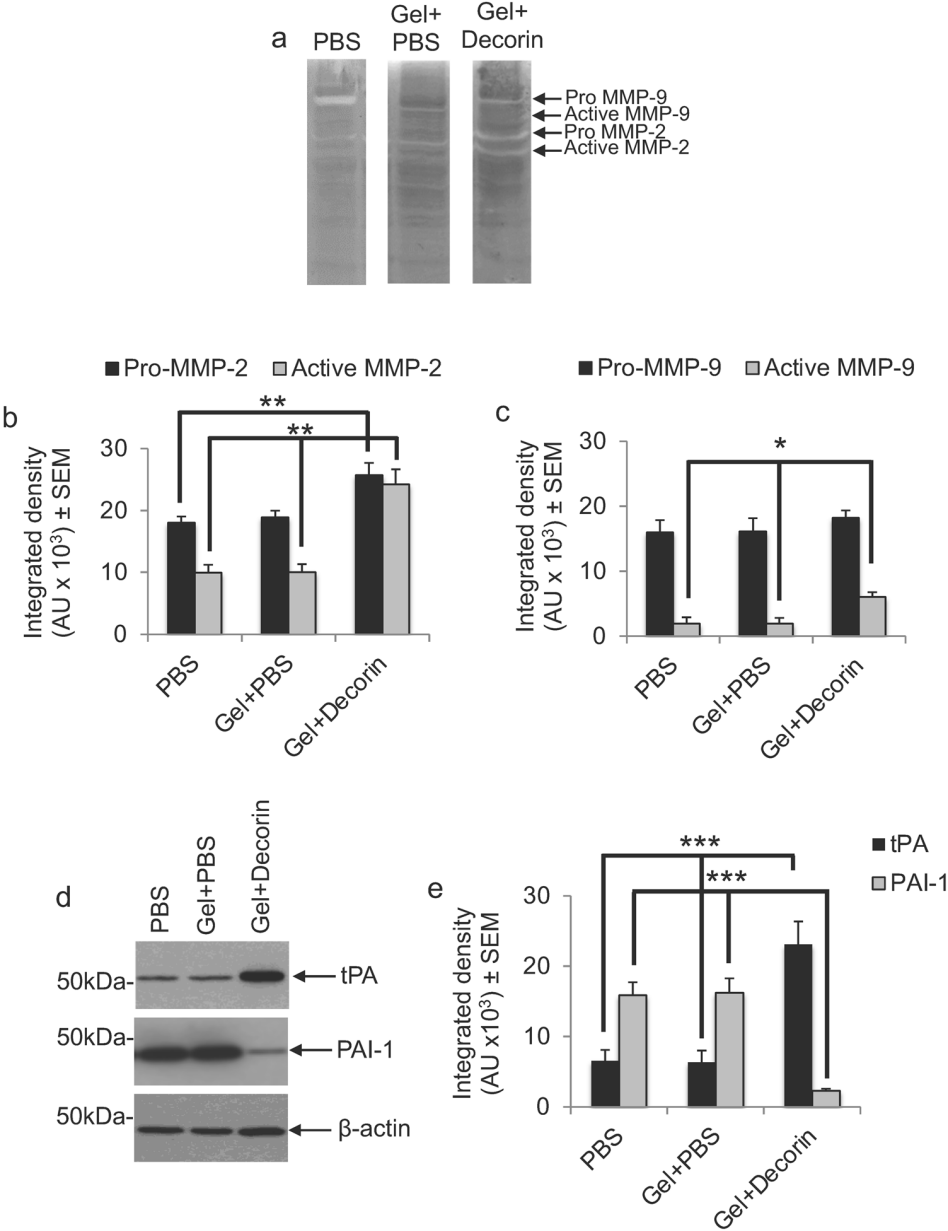
Gel + Decorin enhances MMP and plasminogen activity. (**a**) Zymograms and quantification of (**b**) MMP-2 (pro- and active) and (**c**) MMP-9 (pro- and active) to show enhanced gelatinase activity in Gel + Decorin injected wounds sites compared to wounds treated with Gel + PBS. (**d**) Western blot and subsequent (**e**) quantification to show enhanced tPA, reduced PAI-1 in Gel + Decorin compared to Gel + PBS-treated or PBS alone treated DC wounds. *P < 0.05; **P < 0.001; ***P < 0.0001, one-way ANOVA with Dunnett’s post hoc test. *n* = 4 rats/group, 3 independent repeats, total *n* = 12 rats/group. β-actin was used as a loading control. Original uncropped blots for (a) and (d) are proved as Supplementary Fig. S3 and Supplementary Fig. S4.

Decorin is able to promote axon regeneration by suppressing the production of scar-derived axon growth inhibitory ligands, indirect activation of neurotrophins through the release of plasmin and digestion of CSPG and CNS myelin-derived inhibitors through the release of plasmin (tissue plasminogen activator (tPA)) and activation of MMPs (particularly MMP-2 and MMP-9)^33,34,36^. We therefore determined if DC + Decorin increased tPA levels and MMP activity in DC lesion sites. Zymography and subsequent densitometry using tissues harvested from DC crush sites that were injected with PBS and Gel + PBS, showed no activation of MMP-2 or MMP-9 (Fig. 4a–c). However, in DC crush sites treated with Gel + Decorin, pro-MMP2 levels increased by 1.36-fold, whilst active MMP-2 increased by 2.42-fold compared to Gel + PBS injections (*P* < 0.001, ANOVA; Fig. 4a,b). The levels of pro-MMP-9 remained similar in both groups, however, the levels of active MMP-9 increased 3.22-fold in Gel + Decorin compared to Gel + PBS treatment (*P* < 0.05, ANOVA; Fig. 4a,c). Treatment of DC crush injury sites with Gel + Decorin also significantly enhanced the levels of tPA by 3.65-fold and suppressed levels of PAI-1 by 7.06-fold, compared to PBS and Gel + PBS treatment (*P* < 0.0001, ANOVA; Fig. 4d,e). These results suggest that Gel + Decorin injections locally activate MMP-2 and MMP-9 while enhancing tPA levels and simultaneously suppressing its inhibitor, PAI-1.

### Decorin and Gel + Decorin upregulates regeneration associated genes (RAGs) in vitro and in vivo

**Figure 5:**
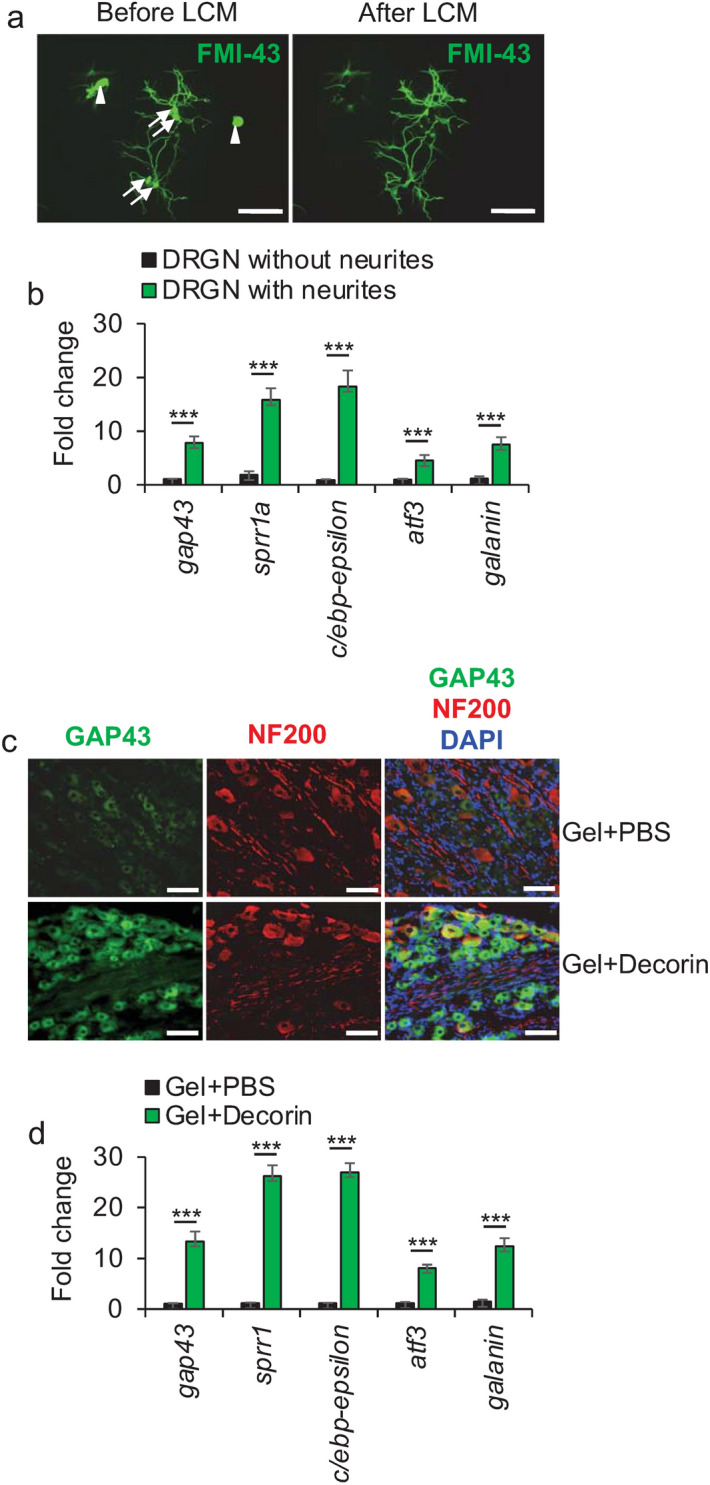
Regeneration associated genes (RAGs) are upregulated in DRGN in response to Decorin treatment. (**a**) LCM to show before and after LCM capture of FMI-43^+^ DRGN cell bodies with (arrows) and without (arrowheads) neurites. **(b**) RAG mRNA expression was significantly higher in DRGN with neurites compared to those without neurites after exposure to Decorin. n = 1000 DRGN/treatment, 6 independent repeats (total *n* = 6000 DRGN/treatment group). (**c**) GAP43 immunohistochemistry to show enhanced levels of GAP43^+^ immunoreactivity in DRGN at 10 days after DC injury and treatment. (**d**) Fold change in RAG mRNA expression levels in L4/L5 dorsal root ganglia (DRG) bundles after Gel + PBS and Gel + Decorin treatment (normalised to mRNA levels in PBS treated wounds). *n* = 8 rats/treatment. Scale bars in (**a**) = 100 µm and (**c**) 25 µm. ***P < 0.0001, one-way ANOVA with Dunnett’s post hoc test.

RAGs such as GAP43, small proline rich protein 1 (sprr1a), c-ebp-epsilon, ATF3 and galanin are normally upregulated in regenerating CNS neurons and increase the intrinsic capacity of the CNS neuron to regenerate^39,40^. Hence, we first used laser capture microdissection (LCM) to harvest cell bodies from DRGN with and without neurites and subjected them to qPCR analysis of RAGs after treatment with Decorin. We demonstrated that all of these RAGs were significantly upregulated in response to Decorin in DRGN with neurites compared to those without neurites (*P* < 0.0001, ANOVA; Fig. 5a,b). Likewise, 10 days after Gel + Decorin treatment in vivo, significant levels of GAP43 immunoreactivity (Fig. 5c) and expression of all of the RAGs were observed, compared to Gel + PBS-treated animals (*P* < 0.0001, ANOVA; Fig. 5d). These results demonstrate that Decorin treatment activates expression of RAGs within DRGN, thereby enhancing their intrinsic capacity to regenerate.

### Gel + Decorin treatment promoted DC axon regeneration in vivo

**Figure 6:**
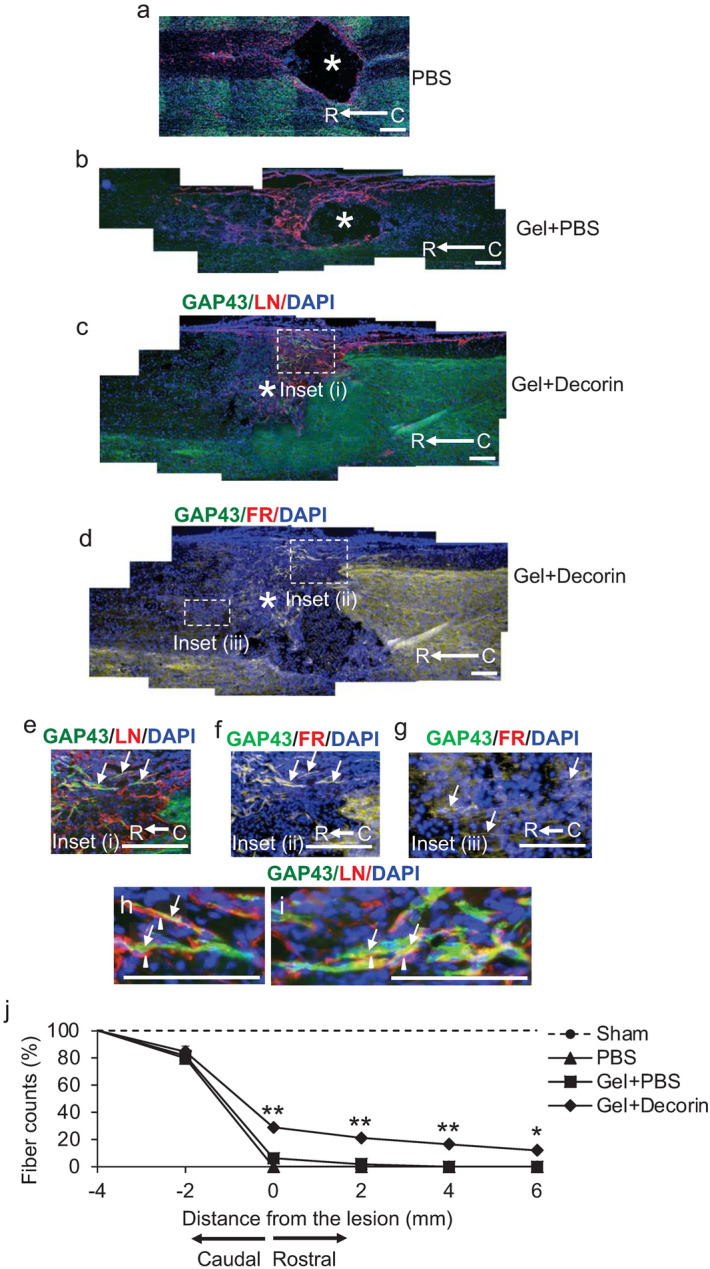
Gel + Decorin promotes DC axon regeneration. Little or no GAP43^+^ (green) axons were localised in (**a**) PBS or (**b**) Gel + PBS injected wounds with a cavity, devoid on LN^+^ immunoreactivity (red), remaining in the lesion core (*). DAPI = nuclear stain (blue). (**c**) No cavity was present in Gel + Decorin injected wounds, with many GAP43^+^ axons (green) traversing the lesion site, which was filled with LN^+^ (red) ECM fibres. (**d**) Merged image from an adjacent section from Gel + Decorin-treated animals costained for FR, GAP43 and DAPI to show overlap of FR and GAP43 staining in the same section. (**e**) High power view of boxed region in (**c**) which is labelled Inset (i) showing GAP43^+^ axons (arrows) regenerating through the lesion site from the caudal (C) to rostral (R) regions of the spinal cord (direction of regeneration is C to R). (**f**) High power view of same boxed region in (c), labelled Inset (ii), which was co-labelled with the bidirectional axon tracer, FluoroRuby (FR), demonstrating overlap of GAP43^+^ with FR^+^ axons (arrows). (**g**) High power view of boxed region in (**c**) which is labelled Inset (iii), showing FR^+^ axons (arrows) that have regenerated into the rostral spinal cord. (**h**) and (**i**) represent high power views GAP43^+^ regenerating axons (arrows), traversing along LN^+^ fibres (arrowheads) within the lesion site of Gel + Decorin-treated animals. (**j**) Quantification of GAP43^+^ axons at different distances from the caudal through the lesion site and in the rostral spinal cord. Scale bars in (**a**–**h**) = 200 µm. ** = P < 0.001, * = P < 0.05, one-way ANOVA with Dunnett’s post hoc test. *n* = 6 rats/group, 3 independent repeats, total *n* = 18 rats/group.

We then determined if Gel + Decorin could facilitate DC axon regeneration. In PBS (Fig. 6a) and Gel + PBS-treated (Fig. 6b) animals, a large cavity remained at the lesion site (*), with few if any GAP43^+^ axons present in rostral or caudal regions adjacent to the DC crush site, with laminin (LN) immunoreactivity being present around the lesion cavity margins. In Gel + Decorin-treated animals, many GAP43^+^ axons were observed crossing the lesion site, rich in LN^+^ ECM fibres, and entering into the distal spinal cord (Fig. 6c,d: inset (i)). Tracing of regenerating axons with FluorRuby (FR) in an adjacent GAP43 immunostained section (individual channels shown in Fig. S2) showed complete overlap in merged images of FR, GAP43 and DAPI (nuclei) in the same section (orange) (Fig. 6d). High power magnifications of the inset (i) in Fig. 6c showed GAP43^+^ axons growing along LN^+^ fibres (arrows; Fig. 6e) whilst high power magnifications from inset (ii) and inset (iii) in Fig. 6d showed GAP43/FR/DAPI merged image demonstrating colocalization of GAP43 and FR in the two regions, respectively (Fig. 6f,g), demonstrating that GAP43 is a good marker of regenerating axons in the rat spinal cord.

In addition, high power photomicrographs showed that GAP43^+^ axons (arrows) within the lesion epicentre were growing along LN^+^ (arrowheads) ECM fibres (Fig. 6h,i). Quantification of the number of GAP43^+^ DC axons regenerating through the lesion site after treatment with Gel + Decorin showed that 29 ± 2, 21.0 ± 1.8, 16.5 ± 1.4 and 12.0 ± 2% of axons regenerated 0, 2, 4 and 6 mm beyond the lesion site, respectively (*P* < 0.001-*P* < 0.05, ANOVA; Fig. 6j). A few GAP43^+^ axons (1.8 ± 0.5%) were also observed in Gel + PBS-treated animals but fell to 0% after 2 mm from the lesion site (Fig. 6j). By contrast, axon numbers in PBS-treated groups fell to 0% at 0 mm from the lesion site and remained at 0% at distances caudal to the lesion (Fig. 6j). These results demonstrate that Gel + Decorin treatment supported significant DC axon regeneration, for up to 6 mm from the lesion site, probably by allowing axons to migrate along LN^+^ ECM fibres deposited within the lesion core.

### Gel + Decorin treatment enhances axon sparing above and below the lesion site

**Figure 7:**
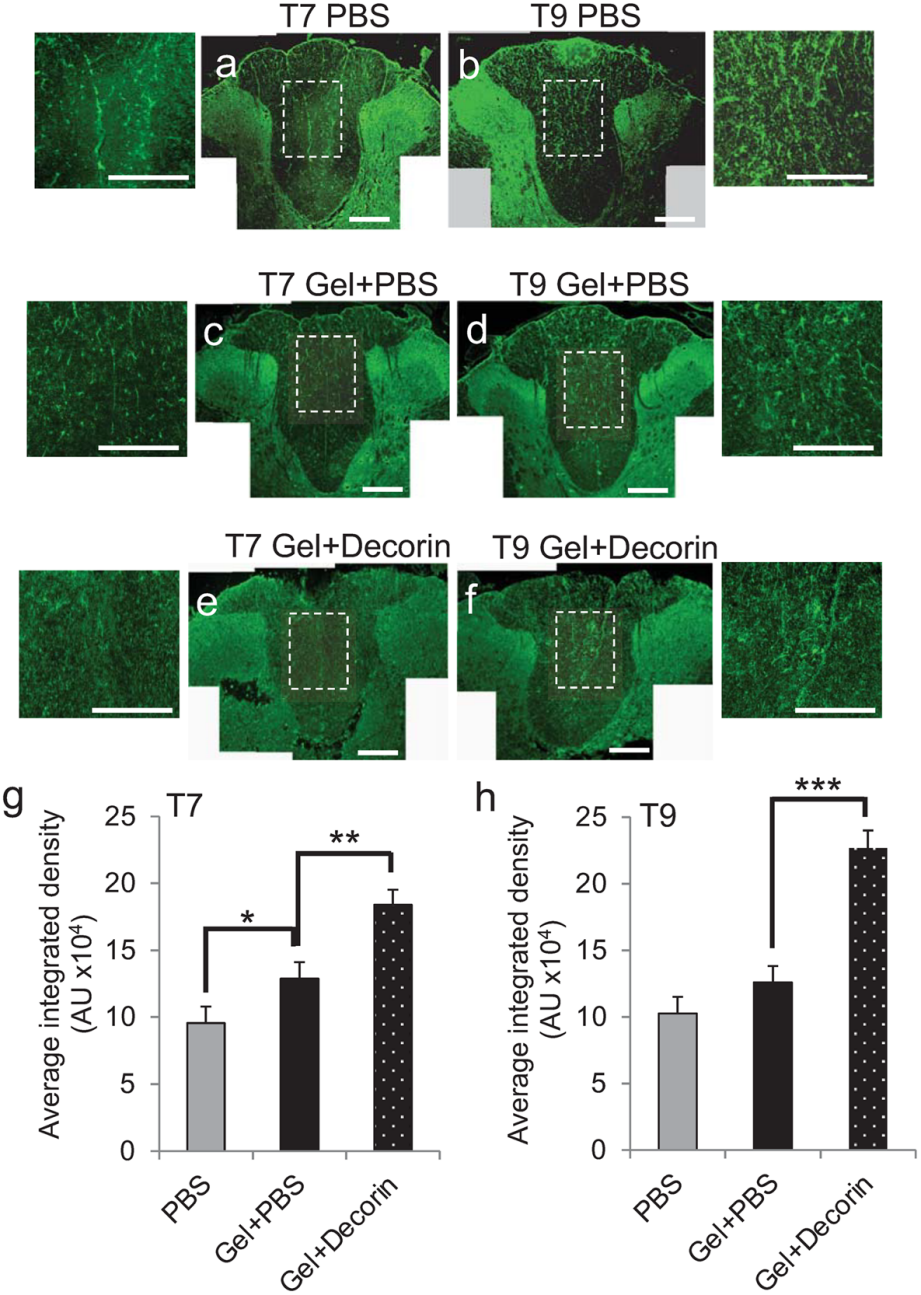
Gel + Decorin promotes sparing of axons above and below the lesion site. NF200^+^ axons after (**a**) and (**b**) PBS treatment, (**c**) and (**d**) Gel + PBS treatment, (**e**) and (**f**) Gel + Decorin in T7 and T9 cord segments, respectively. Quantification of NF200^+^ axons at (**g**) T7 and (**h**) T9 to show significant axon sparing in Gel + Decorin *versus* Gel + PBS treatment above and below the lesion site. Gel + PBS treatment alone also promoted significant sparing below the lesion site at T7, when compared to PBS treatment alone (g). High power insets show more detail of the spared axons in the DC. Scale bars in (**a**)–(**f**) = 200 µm, insets to (**a**)–(**f**) = 100 µm. *P < 0.05; **P < 0.001, **P < 0.0001, one-way ANOVA with Dunnett’s post hoc test. *n* = 4 rats/group, 3 independent repeats, total *n* = 12 rats/group.

Axon sparing/plasticity is important for restitution of functional recovery after DC injury. Therefore, sparing/plasticity of axons in the DC of rats after DC crush injury and Decorin treatment were assessed by calculating the area occupied by NF200^+^ immunoreactivity in the whole dorsal funiculus in tangential cross sections of the spinal cord^41^. DC crush injury sites treated with Gel + Decorin demonstrated significantly greater sparing/plasticity of axons than observed in DC crush injury sites treated with PBS and Gel + PBS, in both above (spinal segment T7) and below the lesion (spinal segment T9) (Fig. 7a–h). NF200^+^ axons were observed in the CST and interneurons and their axons were observed in the superficial dorsal horn (Fig. 7a–f). Quantification of the integrated density of axon staining showed that there was a significant increase in axon sparing/plasticity at T7 but not at T9 in Gel + PBS-treated animals, compared to PBS-treated animals (Fig. 7g,h). However, Gel + Decorin treatment resulted in a significant increase in axon sparing/plasticity in both T7 and T9 compared to Gel + PBS-treated animals (by 1.43- and 1.8-fold in T7 and T9, respectively, when compared to Gel + PBS treatment) (Fig. 7g,h). These results demonstrated that axon sparing/plasticity was significantly enhanced in Gel + Decorin-treated DC crush sites, both above and below the lesion site.

### Gel + Decorin treatment enhanced spinal compound action potentials (CAP) in vivo

**Figure 8:**
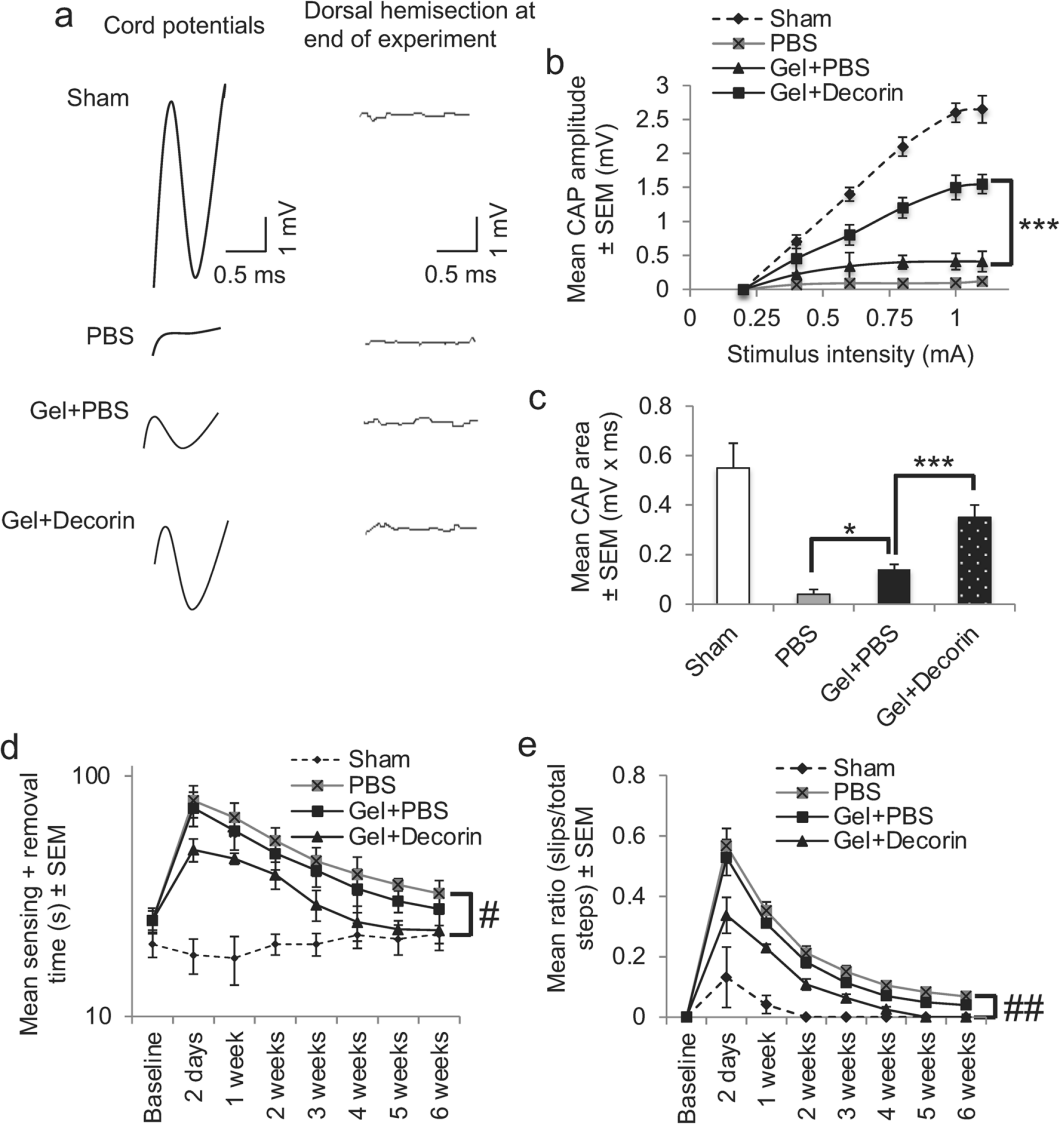
Gel + Decorin injections improved electrophysiological and functional recovery after DC injury. (**a**) Spike 2 processed CAP traces from representative Sham controls, PBS, Gel + PBS and Gel + Decorin-treated rats showing significant recovery of dorsum cord potentials in Gel + Decorin treated rats. There is also a slight improvement in Gel + PBS-treated animals. Dorsal hemisection between the stimulating and the recording electrodes at the end of the experiment ablated CAP traces in all animals demonstrating technical success of the experiment. (**b**) CAP amplitudes (mV) were attenuated in the Gel + PBS treated group but were significantly improved in Gel + Decorin injected rats. (**c**) Mean CAP area was significantly improved in Gel + Decorin *versus* Gel + PBS-treated rats. (**d**) Mean sensing time for the tape sensing and removal test and mean error ratios for the (**e**) horizontal ladder walking test both show significant recovery of sensory and locomotor function, respectively, after Gel + Decorin treatment. *P < 0.05, ***P < 0.0001, one-way ANOVA with Dunnett’s post hoc test; #*P* < 0.0012, linear mixed model (LMM); ##*P* < 0.0001, generalised linear mixed model (GLMM). *n* = 6 rats/group, 3 independent repeats, total *n* = 18 rats/group.

We next determined if the enhanced numbers of GAP43^+^ axons and axon sparing/plasticity translated into improved functional recovery. We first used electrophysiology to determine improvements in CAP traces after PBS, Gel + PBS and Gel + Decorin treatment. The representative CAP traces in Sham treated groups were representative completely attenuated in amplitude after DC injury and PBS treatment (Fig. 8a). A small CAP trace was observed in Gel + PBS-treated animals whereas in Gel + Decorin-treated animals, the amplitude improved markedly (Fig. 8a). The mean CAP amplitude was slightly improved in Gel + PBS-treated animals compared to PBS-treated animals, but were significantly larger in Gel + Decorin-treated animals compared to Gel + PBS treatment (Fig. 8b; *P* < 0.001, ANOVA). The mean CAP area in PBS-treated animals (0.02 ± 0.05 mV x ms) was reduced to 8 ± 0.8% compared to the mean CAP area in Sham-treated groups (0.5 ± 0.08 mV x ms) (Fig. 8c). CAP area in Gel + PBS groups were significantly increased when compared to PBS-treated animals (Fig. 8c; *P* < 0.05). Meanwhile, the mean CAP area in the Gel + Decorin was significantly (*P* < 0.001) larger than in Gel + PBS and was increased to 56 ± 8% of Sham levels (Fig. 8c). These results demonstrate that Gel + Decorin injections promoted the greatest electrophysiological recovery across the lesion site in DC crush injured animals.

### Gel + Decorin treatment enhanced functional recovery

We next determined improvements in sensory and locomotor function using well established and sensitive tests^42–46^. The mean sensing time for the tape sensing and removal test was between 14 and 28 s in Sham-treated animals throughout the 6-week time period (Fig. 8d). A significant increase in sensing time was observed in PBS and Gel + PBS-treated rats, such that at 2 days after DC, it took 79 ± 12 s to detect the tape and remove it (Fig. 8d). Sensing and removal time decreased in the 6-week time period in PBS and Gel + PBS injected animals, with slight improvements in Gel + PBS-treated rats, but remained at around 39–33 s from week 4 onwards. However, in Gel + Decorin-treated animals, sensing time was already significantly less at 2 days after DC injury when compared to PBS or Gel + PBS-treated animals, taking only 49 ± 6 s to detect the adhesive tape. By 4 weeks, there was no significant difference between Gel + Decorin *versus* Sham-treated animals (linear mixed model, *P* < 0.0015).

There was a significant increase in the error rate during horizontal ladder walking, which tests locomotor performance, at 2 days after DC lesion in PBS and Gel + PBS-treated *versus* Sham-treated animals (Fig. 8e). The error rate also increased in Gel + Decorin injected animals compared to Sham-treated animals but the error rate was significantly lower in Gel + Decorin injected *versus* PBs and Gel + PBS-treated animals (Fig. 8e). The mean error rate decreased in PBS, Gel + PBS and Gel + Decorin injected animals but, by 5 weeks after DC lesion, animals treated with Gel + Decorin had returned to Sham treated levels, whilst the error rate remained significantly higher in PBS and Gel + PBS injected animals (generalized linear mixed model, P < 0.0011).

Taken together, these results demonstrate that both sensory and motor function in affected hindlimbs were significantly improved by Gel + Decorin treatment.

## Discussion

The results of this study show that a thermosensitive injectable gel of described physical properties loaded with recombinant human Decorin, sustained the release of significant titres of Decorin over a period of at least 30 days in vitro. In DC crush injured rats, Gel + Gel + Decorin treatment, suppressed cavitation, protected ECM deposition at DC crush wound sites, facilitated repair cell migration, increased RAGs and enhanced DC axon regeneration through the lesion site. Moreover, significant axonal sparing was observed above and below the lesion site in DC crush wounds injected with Gel + Decorin and a significant improvement in DC cord potentials across lesion sites, correlating with improved sensory and motor function in affected hindlimbs in adult rats.

The continued presence of ECM (laminin and fibronectin) in the lesion core of DC crush wounds treated with Gel + Decorin resembles that of the response to SCI in the mouse where post-lesion cavities are normally absent and the lesion core becomes filled with ECM proteins that act as a permissive scaffold for the migration of repair cells^47–50^. This is similar to our previous observations with freeze-dried collagen gels loaded with Decorin where a cavity remained but was significantly smaller when compared to collagen Gel + PBS injected wounds, with no evidence of ECM filled lesion cores^36^. In contrast, the implantation of a gene activated freeze-dried collagen gel into a similar DC lesion site was filled with repair cells, invading astrocytes and regenerating axons without the presence of cavities^51^. This suggests that the responses to an injectable Gel + Decorin treatment are similar to those observed after implantation of freeze-dried collagen gels loaded with Decorin, except that the thermosensitive Gel leads to greater preservation of ECM deposition within the lesion core. This may simply be due to the differences in mechanical properties between freeze-dried gels and the relatively weak thermosensitive Gel used in the current study. It is also likely due to microstructural differences, since collagen-based gels typically exhibit a high-water content (approximately 99%) and are not cross-linked. The current flowable Gel + Decorin treatment allowed injury responsive cells, including astrocytes and macrophages, to migrate into the Gel-filled DC crush wound site and thus promoted DC axon regeneration, presumably through the axogenic properties of Decorin^33,34,36^.

One of the main secondary responses after SCI is the formation of fluid-filled cystic cavities within the glial scar that disperse the scar as they expand and prevent the regeneration of axons, presumably due to a lack of compatible substrates for growth cone attachment^26,47^. This experiment demonstrates that injectable Gel + Decorin treatment promotes cellular ingrowth thereby reducing the formation of secondary cysts that obliterate compatible growth substrates and the fibrotic scar. Implantation of freeze-dried collagen-based gene activated matrices into the DC wound site suppressed cavitation and glial scarring at the lesion site without evidence of axon regeneration^52^, suggesting that filling a DC wound site with collagen hydrogels followed by cellular infiltrate is beneficial in blocking cavitation and fibrosis but is insufficient to enhance axon regeneration. These results support the use of scaffolds made from a variety of products including fibronectin, collagen, hyaluronic acid and hydrogels which are all able to provide cellular support and guidance and reduce cystic cavities in SCI^53–58^.

We also observed that Decorin in vitro and Gel + Decorin treatment in vivo increased the expression of RAGs in regenerating DRGN. Moreover, GAP43 immunoreactivity was also upregulated in DRGN in vivo. Increased expression of RAGs enhances the intrinsic property of CNS neurons to regenerate their damaged axons whilst GAP43 is activated in regenerating axons^39,40^. The enhanced DC axon regeneration observed after Gel + Decorin treatment in vivo is not only likely to be due to the increased expression of RAGs and the properties of the Gel, which acts as a scaffold and a bearer of axon permissive substrates (including laminin) produced by infiltrating repair cells, but may also due to the contribution of Decorin in suppressing/removing axon growth inhibitory ligands within the glial scar^36^. Studies have reported that injection of some matrices supports regeneration of axons into the site of injection by providing a permissive scaffold for regenerating axons to be able to migrate into the lesion site. For example, a polymer-based injectable hydrogel was reported to eliminate cystic cavities by bridging these with FN-rich ECM, and promoting ECM remodelling by activation of MMP-9 thereby promoting functional recovery in adult rats^7^. Thus, all of these effects of the Gel and Decorin combine together to promote significant disinhibited axon regeneration and the eventual restoration of function. The DC injury model is a moderate severity SCI model and it remains to be seen whether filling a rat lesion site with Gel + Decorin in a severe model of SCI will have the same effects that we observed in this study.

Although growth inhibitory elements within fibrotic scars may be detrimental to regenerating axons and their removal may generate a growth permissive environment, the balance of ECM components will determine the potential axon growth response. For example, FN is a well-known substrate for cell adhesion and migration and previous reports have shown that FN matrices can support axon growth in in vivo SCI models^56,59^. Of relevance, FN supports neuroprotection via activation of a multitude of signalling pathways, and thus a fibrotic ECM, which may share some components with a fibrotic scar, may not always be detrimental to axon growth and may promote tissue preservation^7,60,61^. The presence of laminin within the lesion core may also be beneficial in promoting DC axon regeneration since laminin is rich in the ECM of areas of axonal growth in the developing CNS and in areas where axon regeneration is observed including the olfactory system, the optic nerve and in the ventral funiculus^62–65^.

In addition, filling a cavity with ECM and preventing its expansion will protect sensory and motor neuron function by preserving spared axons and their myelination that would be secondarily compromised. Filling the cystic space with ECM minimises neuron/axon exposure to materials in the cystic fluid, which contain increased levels of toxic substances that will contribute to secondary degeneration and demyelination of white matter tracts^7^. Filling the space with a matrix will also stabilise the spinal cord and make it less vulnerable to physical alterations such as tissue collapse^7^. This is evident in the Gel + PBS groups where there is some recovery of function after DC injury. However, Decorin within the Gel also facilitates the clearance of axon growth inhibitory ligands from the DC crush site^34,36^, suppresses astrogliosis, protects against growth cone collapse by inhibition of CSPG signalling, increases integrin expression^66^ and activates neurotrophins by increasing plasmin levels^33^.

Similar to our previous study in acute and chronic DC lesions, Gel + Decorin injections significantly enhanced the activity of MMP-2, MMP-9 and tPA. This permits dissolution of the nascent scar matrix, reduced axon growth inhibitory ligands such as CSPG and NG2, allowing more permissive molecules like laminin to predominate, thus contributing to the promotion of axon regeneration^36^. Decorin, being a natural antagonist against transforming growth factor (TGFβ)1/2 can suppress CNS scarring by functions such as attenuating TGFβ receptor activation and downstream signalling of ECM production, direct binding to type I collagen to inhibit fibrogenesis and activation of plasmin and MMPs to initiate remodelling of wounds^30–32,35,36,67–69^. The presence of GAP43^+^/NF200^+^ axons within the lesion core suggests that the newly filled/remodelled ECM is permissive to axon growth. In addition, an increase in the density of NF200^+^ axons above and below the lesion site directly correlates with significant electrophysiological improvements and improved sensory and locomotor recovery in Gel + Decorin injected animals. Moreover, infiltrating macrophages can also promote regeneration of CNS axons, be it transient, probably through the release of growth promoting factors such as oncomodulin^70–73^.

The model of SCI used by us here is a moderate severity injury and therefore further investigations are required in a severe model of SCI to establish the clinically relevant potential of our Gel + Decorin to promote functional recovery. In addition, our experiments were conducted in a thoracic SCI injury paradaigm which accounts for 35% of all spinal injuries^74^. However, SCI typically affects the cervival level of the spinal cord (50% of all spinal injuries)^74^ and hence SCI models in these regions are also required to establish the clinical relevance of our Gel + Decorin treatment.

In conclusion, our results demonstrate that injectable Gel + Decorin suppresses spinal cord cavitation by allowing the lesion site to become infilled with ECM. Decorin released from the Gel promotes activation of MMPs and increases the expression of RAGs which increase the intrinsic potential of DRGN to regenerate their axons thereby facilitating DC axon regeneration, axonal sparing, and subsequent improvements in sensory and locomotor function. Therefore, our injectable thermosensitive Gel loaded with Decorin has the potential to be an optimal biomaterial for enhancing tissue repair and axon regeneration after SCI.

## Methods

### Preparation of bovine collagen and fibrinogen injectable gels

Bovine collagen (BC: PureCol, Reading, UK) solution (3 mg/ml in HCl, pH 2) was neutralised with potassium orthophosphate (1 M K_2_HPO_4,_ 1 ml: 28 µl) and kept on ice (4 °C). Bovine fibrinogen (FB: Sigma, Poole, UK) solution (4 mg/ml in DMEM) was kept on ice prior to being combined with BC solution. Bovine thrombin (Calbiochem, Watford, UK) solution (200 U/ml in F-12 K) was added to the bovine collagen solution (10 U of thrombin: 4 mg of fibrinogen). The two solutions (BC/FB, 1:3) were mixed immediately prior to injection. Human recombinant Decorin (Decorin: Galacorin™, Catalent Pharma, Somerset, NJ, USA) was concentrated from 5 to 37 mg/ml using Amicon Ultra Centrifugal Filters (Merck, Watford, UK) according to the manufacturer’s instructions, 92.5 µg of Decorin in a total volume of 1.5 µl was added to 3.5 µl BC/FB solution (total volume of gel = 5 µl) and vortexed for 2 s to ensure even mixing.

### Rheology and physical characterisation

Rheology and physical characterisation of the BC/FB Gel was assessed as described previously^75^. Briefly, physical gelation of the BC/FB gel (Gel) was assessed visually. Gels (100 µl) were incubated at 37 °C for 1 h. The BC/FB solution was classed as a Gel if it could be scooped out of the well and did not flow upon agitation, gel formation was also confirmed (G’ >  > G”) using an oscillating rheometer (AR2000). A Smart Swap Peltier plate temperature system with a 40 mm parallel plate was used in all rheological analyses and the temperature was maintained at 37 °C for all tests. Solvent traps were used to ensure that no evaporation or drying took place during each procedure. The oscillation procedure used a conditioning step that applied a pre-shear controlling shear rate of 100 1/s for 30 s. The oscillatory time sweep applied a constant oscillation stress of 0.2 Pa at a frequency of 1 Hz. The oscillatory stress sweep varied the stress from 0.2 Pa until the material yielded at a constant frequency of 1 Hz.

To analyse the viscoelastic properties of the Gel, small deformation oscillatory measurements of storage modulus (G’) and loss modulus (G’’) were taken across a frequency range of 0.1–100 rad s-1 at 37 °C and at a constant strain of 2% (55 mm parallel plate geometry with a 100 mm gap).

### In vitro release of Decorin from the Gel

Pre-made 5 µl Gels containing a total amount of 92.5 µg of Decorin (Galacorin™ from Catalent Pharma Solutions) were incubated at 37 °C in 200 µl artificial cerebrospinal fluid (aCSF; 119 mM NaCl, 26.2 mM NaHCO_3_, 1 mM NaH_2_PO_4_, 1.3 mM MgCl_2_ and 10 mM glucose, pH 7.4). All plastics were pre-coated with bovine serum albumin (BSA; Sigma) to minimise protein adhesion. The complete volume of aCSF (200 µl) was collected from each sample at specified times (12 h, 24 h, 48 h, 7 days and 15 days) and replaced with 200 µl of fresh aCSF. The concentration of Decorin in each collected sample was measured using enzyme-linked immunosorbent assay (ELISA) kit (R&D Systems, Abingdon, UK), according to the manufacturer’s instructions. At the end of the experiment, gels were mechanically disrupted in Eppendorf tubes, clarified by centrifugation at 13,000 × *g* for 20 min and the supernatant was also put through an ELISA to determine the amount of Decorin retained within the gel.

### Animals

We used adult female Sprague–Dawley rats (Charles River, Maidstone, UK) weighing 170–220 g for all experiments in this study. Animals were fed a commercially available diet and water ad libitum under controlled conditions (22 ± 2 °C, 55% ± 5% humidity, and a 12-h light/12-h dark cycle).

All animal experiments were licensed by the UK Home Office and experimental protocols were approved by the University of Birmingham’s Animal Welfare and Ethical Review Board. Animal surgeries were carried out in strict accordance to the guidelines of the UK Animals Scientific Procedures Act, 1986 and the Revised European Directive 1010/63/EU and conformed to the ARRIVE guidelines and the recommendation of the use of animals by the Federation of the European Laboratory Animal Science Associations. After surgery, animals were returned to their home cages and pre- and post-operative analgesia (Buprenrophine) was provided as standard and with guidance from the named veterinary surgeon.

In vivo sample sizes were determined at the outset and were based on power calculations derived from previous similar experiments in our laboratories using the NC3Rs Experimental Design Assistant (https://www.nc3rs.org.uk/experimental-design-assistant-eda). Animals were randomly assigned to experimental groups using the ‘block randomisation’ method, with experimenters masked to the treatment and procedural conditions. No animals were excluded from this study and no expected or unexpected adverse effects were encountered. Animal tissue samples were all processed and analyzed together to prevent batch effects. All animals were housed in groups of four animals/cage in the same facility and the number of biological replicates is indicated in the figure legends. Treatments were administered at the same time each day and in the same order. Humane endpoints included body weight loss of > 20%, autophagia of the paralysed/insensitive limbs such that haemorrhage or removal of the outside digit distal phalange had occurred. However, none of the animals suffered any of these adverse events and hence no animals were excluded from the study.

### Serum withdrawal model of adult rat dorsal root ganglion neurons

Primary rat dorsal root ganglion neurons (DRGN) were prepared from 6–8-week-old adult Sprague–Dawley rats (weighing 170–220 g; Charles River) as described by us previously ^38,76^. Briefly, animals were killed by rising concentrations of CO_2_, the L4-L7 DRG pairs were dissected out and dissociated into single cells using Neurobasal-A (Invitrogen, Paisley, UK) containing 0.1% collagenase (Sigma, Poole, UK). To mimic a serum withdrawal model to assess DRGN survival, dissociated DRGN were pelleted and resuspended in Dulbecco’s Modified Eagle’s Medium (DMEM; Invitrogen) containing 5-Fluoro-2-deoxyuridine (to limit glial proliferation) at a final concentration of 30 µM. DRGN were plated at a density of 500 cells/well in 8-well chamber slides (BD Biosciences, Oxford, UK) pre-coated with 100 µg/ml poly-D-lysine and 20 µg/ml Laminin-I (both from Sigma) and incubated for 5 days in a humidified chamber set at 37 °C and 5% CO_2_. Experiments were performed in duplicate and repeated on 3 independent occasions (*n* = 6 wells/condition).

### Laser capture microdissection (LCM) of DRGN with and without neurites

Laser capture microdissection of DRGN with and without neurites was performed as described by others and us^77,78^. Briefly, dissociated DRGN were stained with fluorescent lipophilic dye, FMI-43 [N-(3- triethylammoniumpropyl)-4-4-(4-(dibutylamino)styryl)pyridinium dibromide] (Invitrogen) for 20 min^77,78^, fixed with 4% paraformaldehyde and 4% sucrose in PBS for 10 min, dehydrated through a graded series of ethanols and air-dried. An LCM microscope (Arcturus Pixcell II; Applied Biosystems, Rugby, UK) was used to isolate 1000 FMI-43^+^ DRGN with and without neurites in each experiment and the RNA extracted, amplified, and quantitative (q)RT-PCR performed as described below. Experiments were performed in duplicates and repeated on three independent occasions (total *n* = 6).

### Quantitative-RT-PCR analysis (qRT-PCR)

Selected mRNA was validated by qRT-PCR after preparing complementary DNA from extracted RNA and qRT-PCR was performed using a Light-Cycler real time qRT-PCR machine (Roche, Burgess Hill, UK) according to previously published methods^78,79^. Fold changes in mRNA were calculated using the ΔΔCt method^78,79^. qPCR primer sets for regeneration associated genes (RAGs) were purchased from ThermoFisher Scientific and included: *gap43* (Cat no. 4331182, Reaction ID. Rn00567901_m1), *sprr1a* (Cat no. 4331182, Reaction ID. Rn02061965_s1), *c/ebp-epsilon* (Cat no. 4331182, Reaction ID. Rn00567306_g1), *atf3* (Cat no. 4331182, Reaction ID. Rn00563784_m1) and *galanin* (Cat no. 4331182, Reaction ID. Rn00583681_m1).

### Immunocytochemistry

DRGN were fixed in 4% (*v/v*) paraformaldehyde (TAAB Laboratories, Berkshire, UK), washed × 3 in PBS, blocked in PBS containing 0.1% Triton X-100 and incubated with monoclonal anti-βIII-tubulin antibodies (Sigma; diluted at 1:200), to detect DRGN soma and neurites, as described previously^38,76^. DRGN were then washed in PBS and incubated secondary antibodies labelled with Alexa-Fluor 488 (Invitrogen). After final washes in PBS, chambers were removed and coverslips mounted with Vectamount containing DAPI (Vector Laboratories, Peterborough, UK). Cells were viewed, by an observer masked to the treatment conditions, under a Zeiss epi-fluorescent microscope equipped with an Axiocam HRc and running Axiovision Software (all from Zeiss, Hertfordshire, UK). Negative controls including omission of primary antibody were included in all experiments and were used to set the background threshold prior to image capture.

### Quantification of DRGN survival and neurite outgrowth

DRGN survival and neurite lengths were determined by a blinded observer, as described by us previously^38,76^. Briefly, DRGN survival was quantified by dividing each 8-well chamber into 9 quadrants and counting the number of βIII-tubulin^+^ DRGN with DAPI^+^ nuclei in random photomicrographs from each quadrants (n = 54 fields/condition). The number of DRGN with neurites were quantified from the same photomicrographs and were recorded only if their neurite projected greater than 20 µm from the cell soma^76^.

To quantify DRGN neurite outgrowth, the longest βIII-tubulin^+^ neurite emanating from 30 randomly selected DRGN/coverslip were measured using the built-in measurement facilities in Axiovision^76^.

### In vivo experiments

#### In vivo experimental design

With the experimenter blinded to the treatment conditions, animals were randomly assigned and divided into five groups comprising: (1), Control intact animals (Intact); (2), Sham surgery (baseline controls; exposure of the spinal cord after a partial laminectomy but no DC crush (Sham); (3) DC crush + 5 µl PBS (PBS); (4), DC crush + 3.5 µl Gel + 1.5 µl PBS (Gel + PBS) and (5), DC crush + 3.5 µl Gel + 1.5 µl Decorin (Gel + Decorin), each consisting of *n* = 6 animals/group and repeated on 3 independent occasions (total *n* = 18 rats/group). Functional recovery was assessed at baseline, 2 days and weekly for 6 weeks, whilst electrophysiology was performed prior to killing the same animals. After undergoing functional recovery tests and electrophysiology animals were killed by overdose of anaesthesia (Isofluorane) followed by intracardiac perfusion with 4% paraformaldehyde and tissues were harvested for histology, as described below. Separate animals were used for western blot (n = *4* rats/group, 3 independent repeats, total *n* = 12 rats/group) and zymography (*n* = 4 rats/group, 3 independent repeats, total *n* = 12 rats/group) and were killed by rising concentrations of CO_2_ at 6 weeks after DC injury and treatment.

### Animal surgery

DC crush was performed in adult female Sprague–Dawley rats as described by us previously^36,47^. Briefly, animals were injected with Buprenorphine before anaesthesia using 5% Isofluorane and 1.8 l/min O_2_ and a maintenance dose of 2% isofluorane/1.8 l/min O_2_. A partial laminectomy was performed to expose the spinal cord and the DC were crushed bilaterally at the level of T8 using a calibrated watchmaker’s forceps. The crush site was immediately injected with vehicle PBS (5 µl) or 3.5 μl of BC/FB solution and 1.5 µl of PBS, or 3.5 μl of BC/FB + 1.5 µl of Decorin solution, containing a total of 92.5 μg of Decorin, which instantly formed a gel in the lesion site. Control crush sites were injected with PBS alone. Animals were allowed to recover for 6 weeks after DC crush, prior to being subjected to functional testing at 2 and 7 days and then weekly for 6 weeks and electrophyisology at 6 weeks, as described below.

### FluoroRuby injections

The bidirectional tracer, FluoroRuby (FR: MW 10,000; ThermoFisher Scientific, Leicester, UK) was injected slowly at a depth of 0.5 mm, 10 mm caudal to the lesion site 1 day before animal sacrifice, using a micropipette with a tip diameter between 30 and 40 µm^80^. Rats were then intracardially perfused with 4% paraformaldehyde, cryoprotected in sucrose, embedded in optimal cutting temperature (OCT; Raymond A Lamb, Peterborough, UK) embedding medium and longitudinal sections of the spinal cord cut at a thickness of 15 µm using a cryostat, as described in the immunohistochemistry section below.

### Tissue preparation and immunohistochemistry

The DC crush site and tissue 8 mm either side of the crush site were dissected out, post-fixed in 4% paraformaldehyde for 2 h, washed in 3 changes of PBS, cryoprotected in a graded series of sucrose solutions and embedded in OCT embedding compound (TAAB Laboratories, Peterborough, UK)^47^. Para-sagittal (*n* = 4 rats/group, 3 independent repeats, total *n* = 12 rats/group) or cross sections (*n* = 4 rats/group, 3 independent repeats, total *n* = 12 rats/group), for GAP43/collagen-1/laminin/ED1 and GFAP or NF200 immunoreactivity respectively, of the spinal cord were cut at 15 µm-thick using a Bright’s Instrument cryostat (Cambridge, UK) and adhered onto charged Superfrost slides (Fisher Scientific, Loughborough, UK) and stored at -20 °C until required.

Immunohistochemistry was performed as described by us previously^47^. Briefly, sections were selected from either side of the centre of the lesion and thawed at room temperature, permeabilised in 0.1% Triton X-100 (Sigma) and incubated overnight (16–18 h) in primary antibody (Table 1) in a humidified chamber. Sections were washed in several changes of PBS and incubated with either Alexa 488 or Alexa 594-labelled secondary antibodies (Table 1), for 1 h at room temperature. After final washes in PBS, coverslips were then mounted using Vectashield containing DAPI (Vector Laboratories). Negative controls, including omission of primary antibody, were included in each run and were used to set the background threshold for image capture using an Axioplan-2 epi-fluorescent microscope equipped with an Axiocam HRc and controlled by Axiovision software, version 4.0 (all purchased from from Zeiss, Hertfordshire, UK).

**Table 1:** List of primary and secondary antibodies used in this study.

Antibody	Source	Dilution	
		IHC	WB
**Primary antibodies**			
Rabbit anti-collagen I	Abcam, Cambridge, UK	1:500	
Rabbit anti-laminin	Sigma, Poole, UK	1:400	
Mouse anti-ED1	Serotec, Kidlington, UK	1:200	
Mouse anti GFAP	Sigma, Poole, UK	1:400	
Mouse anti-GAP43	Invitrogen, Paisley, UK	1:400	
Mouse anti-NF200	Sigma, Poole, UK	1:400	
Rabbit anti-tPA	Abcam, Cambridge, UK		1:500
Rabbit anti-PAI-1	Abcam, Cambridge, UK		1:500
Mouse anti-β-actin	Sigma, Poole, UK		1:1000
**Secondary antibodies**			
Alexa488-anti-rabbit IgG	Invitrogen, Paisley, UK	1:400	
Alexa488 anti-mouse IgG	Invitrogen, Paisley, UK	1:400	
Alexa594 anti-mouse IgG	Invitrogen, Paisley, UK	1:400	
Alexa594 anti-rabbit IgG	Invitrogen, Paisley, UK	1:400	
HRP anti-rabbit IgG	GE Healthcare, Hertfordshire, UK		1:1000
HRP anti-mouse IgG	GE Healthcare, Hertfordshire, UK		1:1000

*IHC* immunohistochemistry, *WB* western blot.

### Tissue lysis and western blot

The DC crush site and tissue to 3 mm either side of the crush site from n = 4 rats/group (3 independent repeats, total *n* = 12 rats/group) were pooled together and subjected to tissue lysis and western blot analysis, as described previously^47^. Briefly, tissues were homogenised in ice-cold lysis buffer containing 20 mM Tris HCl, 1% NP-40, 3 mM EDTA and 3 mM EGTA and clarified by centrifugation at 13,000 rpm. Protein content was determined using a Biorad DC protein assay kit and 40 μg of total protein was resolved on 12% SDS-PAGE gels. Proteins were then transferred to PVDF membranes (Millipore, Watford, UK) and probed with rabbit anti-tPA (tissue plasminogen activator) and rabbit anti-PAI-1 (plasminogen activator inhibitor-1) antibodies (Table 1, Abcam, Cambridge, UK). Bands were detected with HRP-labelled anti-rabbit IgG (Table 1, GE Healthcare, Buckingham, UK) and visualised with an enhanced chemiluminescence kit according to the manufacturer’s instructions (GE Healthcare). Luminescent bands were scanned using Adobe Photoshop and densitometrically analysed using the gel plotting macros in Image J (NIH Image, Bethesda, USA).

### Zymography

Zymography was used to analyse gelatinase activity in DC crush sites from PBS-, Gel + PBS- and Gel + Decorin-treated animals, based on previously published methods^36,47,81^. Briefly, the DC crush site and tissue to 3 mm either side of the lesion site from *n* = 4 rats/group (3 independent repeats, total *n* = 12 rats/group) were pooled together, homogenised without protease inhibitors and 40 μg of total protein was resolved on 10% minigels incorporated with gelatin substrates (Invitrogen, Paisley, UK) and clear protein lysis bands on a Coomassie Blue stained background were visualised according to the manufacturer’s instructions (Invitrogen). Bands were scanned using Adobe Photoshop and densitometry was performed in ImageJ (NIH Image) using the built-in gel plotting macros.

### Lesion cavity size

The middle three sections through each lesion cavity, stained with GFAP were used to measure lesion cavity area using ImagePro Analyzer (*n* = 4 rats/group, 3 independent repeats (total *n* = 12 rats/group)), as described by us previously^47^.

### Quantification of spared axons/plasticity

Cross-sections of the spinal cord (*n* = 4 rats/group, 3 independent repeats, total *n* = 12 rats/group) above and below the lesion site (i.e. T7 and T9) were stained with neurofilament 200 (NF200) antibodies (Table 1) to quantify axonal sparing in the DC, as described previously^47^. Briefly, images captured on an Axioplan-2 epi-fluorescent microscope equipped with an Axiocam HRc (Zeiss) were analysed in ImageJ (NIH Image) and the relative intensity of NF200^+^ immunoreactivity was measured in the traced area of the entire dorsal funiculus after normalisation of the background intensity. Automatic thresholding was then used to determine pixel intensities above threshold levels and presented graphically.

### Quantification of DC axon regeneration

Regeneration of axons in the DC was quantified by GAP43 immunohistochemistry, as previously described^82^. GAP43 was used as a marker of regenerating axons since, in our hands, cholera toxin B labelling does not detect regenerating axons in the rat^36^. However, we also used the bidirectional tracer FR to trace axons in the same sections as GAP43 immunohistochemistry to confirm that GAP43 labelled regenerating axons, since FR labelling overlayed GAP43 immunoreactivity. Briefly, serial parasagittal sections of cords were reconstructed by collecting all serial 50 μm-thick sections (~70–80 sections/cord; *n* = 4 rats/treatment, 3 independent repeats; total *n* = 12 rats/treatment group). In each section, the numbers of intersections of GAP43^+^ fbers through a dorsoventral orientated line was counted from 4 mm rostral to 6 mm caudal to the lesion site. Axon number was calculated as a percentage of the fibers seen 4 mm above the lesion, where the DC was intact. The measured distance beyond the epicenter of the lesion towards the rostral end was scored as a negative number and towards the caudal end as a positive number.

### Functional tests

Functional tests to monitor locomotor and sensory function in Sham animals and after DC lesion and PBS-, Gel + PBS- or Gel + Decorin-treatment, was carried out as described by others^42^. Animals (*n* = 6/treatment group, 3 independent repeats; total *n* = 18 rats/treatment group) were trained for 2 weeks prior to functional testing and had mastered traversing the rope and ladder. To establish baseline parameters, all functional tests were performed 2–3 days before DC injury. Animals were then tested 2 days after DC injury and then weekly for 6 weeks. Experiments were performed by 2 observers masked to treatment conditions, in the same order, at the same time of day and each test was performed for 3 individual trials. Animals that mis-stepped or ‘stuttered’ were counted as slips.

#### Horizontal ladder test (locomotor function)

Animals were allowed to traverse a 0.9-m-long horizontal ladder with a diameter of 15.5 cm and randomly adjusted rungs with possible gaps of 3.5–5.0 cm to assess locomotion across the ladder^42^. The left and right rear paw slips were recorded and the total number of slips was divided by the total number of steps to calculate the mean error ratio.

#### Tape sensing and removal test (sensory function)

Animals were held with both hind-paws extended and a piece of tape of 15 × 15 mm (Kip Hochkrepp, Bocholt, Germany) was affixed to the palm of the left hind-paw. The time it took for the animals to detect the tape was recorded and used to calculate the mean sensing time.

### Electrophysiological recordings

At 6 weeks after surgery, treatment, and functional recovery tests, compound action potentials (CAP) were recorded as previously described^83,84^. The experimenter was blinded to the treatment status of the animals (same animals as tape sensing/removal and locomotor tests as described above used). Briefly, Sham-treated animals and animals after DC and PBS, Gel + PBS and Gel + Decorin treatment (*n* = 6 rats/treatment group, 3 independent repeats; total *n* = 18 rats/treatment group) were deeply anesthetised under Isofluorane and maintained at 1% Isofluorane concentration throughout the recording process. Silver wire electrodes (0.01 diameter) insulated except at the tip were used to stimulate the DC axons at L1-L2 and recording of CAP at T10-T9 at the surface of the midline spinal cord. Pancuronium bromide (0.3 mg/kg, Sigma) was injected intraperitoneally to minimize muscular contractions throughout the experiment. The signal from the recording electrode was amplified with filters set at 300–3000 Hz (DAM 80, WPI, Sarasota, FL, USA) and Spike 2 software (Cambridge Electronic Design, Cambridge, UK) was used to analyse the data. Single current pulses (0.05 ms) were applied through a stimulus isolation unit (A365, WPI and Master-8, from A.M.P.I., Jerusalem, Israel) in increasing increments (0.2, 0.3, 0.6, 0.8, 1.1 mA). The CAP amplitude was calculated as the value between the negative deflection after the stimulus artifact and the next peak of the wave. The area of the CAP was calculated by rectifying the CAP component (full-wave rectification) and measuring its area. At the end of each experiment, the dorsal half of the spinal cord was transected between stimulating and recording electrodes to confirm that a CAP could not be detected.

### Statistical analyses

All data are presented as means ± SEM. Tests for normality were performed in SPSS, Version 24 (IBM Corporation, Armonk, NY, USA) using the Shapiro–Wilk test, since smaples sizes were < 50. Parametric data was then analysed in SPSS using one-way analysis of variance (ANOVA) assuming unequal variances. Post hoc tests with Dunnett’s method were used to test for statistical differences between means.

For the ladder crossing functional test, data was analysed according to published methods^42,43^ using R package (www.r-project.org). Briefly, whole time-course of lesioned and sham-treated animals were compared using binomial generalized linear mixed models (GLMM), with: lesion/sham set to true in lesioned animals post-surgery (set to false otherwise); operated/unoperated set to false before surgery, true after surgery as fixed factors; animals as random factors and time as a continuous covariate. Binomial GLMMs were fitted in R using *lme4* with the *glmer* functions. P values were then calculated using parametric bootstrap. For the tape removal test, linear mixed models (LMM) were calculated by model comparison using the package *pbkrtest*, with the Kenward-Roger method^42,43^. Independent sample t-test were performed in SPSS (IBM) to determine differences between individual points in the tape removal and ladder walking tests.

## Supplementary Information

Supplementary Figures.

## Data Availability

The data that support the findings of this study are available from the corresponding author upon reasonable request.

## References

[1] Rolls A, Shechter R, Schwartz M (2009). The bright side of the glial scar in CNS repair. Nat. Rev. Neurosci..

[2] Straley KS, Foo CW, Heilshorn SC (2010). Biomaterial design strategies for the treatment of spinal cord injuries. J. Neurotrauma.

[3] Pires LR, Pego AP (2015). Bridging the lesion-engineering a permissive substrate for nerve regeneration. Regen. Biomater..

[4] Perale G (2011). Hydrogels in spinal cord injury repair strategies. ACS Chem. Neurosci..

[5] Perale G (2012). Multiple drug delivery hydrogel system for spinal cord injury repair strategies. J. Control. Release.

[6] Libro R, Bramanti P, Mazzon E (2017). The combined strategy of mesenchymal stem cells and tissue-engineered scaffolds for spinal cord injury regeneration. Exp. Ther. Med..

[7] Hong LTA (2017). An injectable hydrogel enhances tissue repair after spinal cord injury by promoting extracellular matrix remodeling. Nat. Commun..

[8] Corey JM (2007). Aligned electrospun nanofibers specify the direction of dorsal root ganglia neurite growth. J. Biomed. Mater. Res. A.

[9] De Laporte L, Yan AL, Shea LD (2009). Local gene delivery from ECM-coated poly(lactide-co-glycolide) multiple channel bridges after spinal cord injury. Biomaterials.

[10] Machado HA, Abercrombie JJ, You T, Deluca PP, Leung KP (2013). Release of a wound-healing agent from PLGA microspheres in a thermosensitive gel. Biomed. Res. Int..

[11] Tysseling-Mattiace VM (2008). Self-assembling nanofibers inhibit glial scar formation and promote axon elongation after spinal cord injury. J. Neurosci..

[12] Zuidema JM (2014). Enhanced GLT-1 mediated glutamate uptake and migration of primary astrocytes directed by fibronectin-coated electrospun poly-L-lactic acid fibers. Biomaterials.

[13] Cheng H, Huang YC, Chang PT, Huang YY (2007). Laminin-incorporated nerve conduits made by plasma treatment for repairing spinal cord injury. Biochem. Biophys. Res. Commun..

[14] Matsumoto K (2000). Use of a newly developed artificial nerve conduit to assist peripheral nerve regeneration across a long gap in dogs. ASAIO J..

[15] Wilcox JT, Cadotte D, Fehlings MG (2012). Spinal cord clinical trials and the role for bioengineering. Neurosci. Lett..

[16] King VR, Alovskaya A, Wei DY, Brown RA, Priestley JV (2010). The use of injectable forms of fibrin and fibronectin to support axonal ingrowth after spinal cord injury. Biomaterials.

[17] Petter-Puchner AH (2007). The long-term neurocompatibility of human fibrin sealant and equine collagen as biomatrices in experimental spinal cord injury. Exp. Toxicol. Pathol..

[18] Cheng H, Cao Y, Olson L (1996). Spinal cord repair in adult paraplegic rats: Partial restoration of hind limb function. Science.

[19] Hyatt AJ, Wang D, Kwok JC, Fawcett JW, Martin KR (2010). Controlled release of chondroitinase ABC from fibrin gel reduces the level of inhibitory glycosaminoglycan chains in lesioned spinal cord. J. Control. Release.

[20] Marchand R, Woerly S (1990). Transected spinal cords grafted with in situ self-assembled collagen matrices. Neuroscience.

[21] Gelderd JB (1990). Evaluation of blood vessel and neurite growth into a collagen matrix placed within a surgically created gap in rat spinal cord. Brain Res..

[22] Joosten EA, Bar PR, Gispen WH (1995). Collagen implants and cortico-spinal axonal growth after mid-thoracic spinal cord lesion in the adult rat. J. Neurosci. Res..

[23] Gupta D, Tator CH, Shoichet MS (2006). Fast-gelling injectable blend of hyaluronan and methylcellulose for intrathecal, localized delivery to the injured spinal cord. Biomaterials.

[24] Baumann MD, Kang CE, Tator CH, Shoichet MS (2010). Intrathecal delivery of a polymeric nanocomposite hydrogel after spinal cord injury. Biomaterials.

[25] Lee H, McKeon RJ, Bellamkonda RV (2010). Sustained delivery of thermostabilized chABC enhances axonal sprouting and functional recovery after spinal cord injury. Proc. Natl. Acad. Sci. U. S. A..

[26] Lagord C, Berry M, Logan A (2002). Expression of TGFbeta2 but not TGFbeta1 correlates with the deposition of scar tissue in the lesioned spinal cord. Mol. Cell. Neurosci..

[27] Silver JR (2004). Medical cases who would benefit from treatment on a spinal injury unit. Spinal Cord.

[28] Logan A (1994). Effects of transforming growth factor beta 1 on scar production in the injured central nervous system of the rat. Eur. J. Neurosci..

[29] Zhang JM, Hoffmann R, Sieber-Blum M (1997). Mitogenic and anti-proliferative signals for neural crest cells and the neurogenic action of TGF-beta1. Dev. Dyn..

[30] Davies JE, Tang X, Denning JW, Archibald SJ, Davies SJ (2004). Decorin suppresses neurocan, brevican, phosphacan and NG2 expression and promotes axon growth across adult rat spinal cord injuries. Eur. J. Neurosci..

[31] Hocking AM, Shinomura T, McQuillan DJ (1998). Leucine-rich repeat glycoproteins of the extracellular matrix. Matrix Biol..

[32] Logan A, Baird A, Berry M (1999). Decorin attenuates gliotic scar formation in the rat cerebral hemisphere. Exp. Neurol..

[33] Davies JE, Tang X, Bournat JC, Davies SJ (2006). Decorin promotes plasminogen/plasmin expression within acute spinal cord injuries and by adult microglia in vitro. J. Neurotrauma.

[34] Minor K (2008). Decorin promotes robust axon growth on inhibitory CSPGs and myelin via a direct effect on neurons. Neurobiol. Dis..

[35] Reese SP, Underwood CJ, Weiss JA (2013). Effects of decorin proteoglycan on fibrillogenesis, ultrastructure, and mechanics of type I collagen gels. Matrix Biol..

[36] Ahmed Z (2014). Decorin blocks scarring and cystic cavitation in acute and induces scar dissolution in chronic spinal cord wounds. Neurobiol. Dis..

[37] Silva D, Sousa RA, Salgado AJ (2021). Hydrogels as delivery systems for spinal cord injury regeneration. Mater Today Bio.

[38] Vigneswara V, Berry M, Logan A, Ahmed Z (2013). Pigment epithelium-derived factor is retinal ganglion cell neuroprotective and axogenic after optic nerve crush injury. Invest Ophthalmol. Vis. Sci..

[39] Fawcett JW (2020). The struggle to make CNS axons regenerate: Why has it been so difficult?. Neurochem. Res..

[40] Qian C, Zhou FQ (2020). Updates and challenges of axon regeneration in the mammalian central nervous system. J. Mol. Cell Biol..

[41] Bouhy D (2011). Inhibition of the Ca(2)(+)-dependent K(+) channel, KCNN4/KCa3.1, improves tissue protection and locomotor recovery after spinal cord injury. J. Neurosci..

[42] Fagoe ND (2016). Evaluation of five tests for sensitivity to functional deficits following cervical or thoracic dorsal column transection in the rat. PLoS ONE.

[43] Almutiri S, Berry M, Logan A, Ahmed Z (2018). Non-viral-mediated suppression of AMIGO3 promotes disinhibited NT3-mediated regeneration of spinal cord dorsal column axons. Sci. Rep..

[44] Farrukh F, Davies E, Berry M, Logan A, Ahmed Z (2019). BMP4/Smad1 signalling promotes spinal dorsal column axon regeneration and functional recovery after injury. Mol. Neurobiol..

[45] Stevens AR, Ahmed U, Vigneswara V, Ahmed Z (2019). Pigment epithelium-derived factor promotes axon regeneration and functional recovery after spinal cord injury. Mol. Neurobiol..

[46] Tuxworth RIT (2019). Attenuating the DNA damage response to double-strand breaks restores function in models of CNS neurodegeneration. Brain Commun..

[47] Surey S, Berry M, Logan A, Bicknell R, Ahmed Z (2014). Differential cavitation, angiogenesis and wound-healing responses in injured mouse and rat spinal cords. Neuroscience.

[48] Inman DM, Steward O (2003). Physical size does not determine the unique histopathological response seen in the injured mouse spinal cord. J. Neurotrauma.

[49] Sroga JM, Jones TB, Kigerl KA, McGaughy VM, Popovich PG (2003). Rats and mice exhibit distinct inflammatory reactions after spinal cord injury. J. Comp. Neurol..

[50] Byrnes KR, Fricke ST, Faden AI (2010). Neuropathological differences between rats and mice after spinal cord injury. J. Magn. Reson. Imaging.

[51] Gonzalez AM, Berry M, Greenlees L, Logan A, Baird A (2006). Matrix-mediated gene transfer to brain cortex and dorsal root ganglion neurones by retrograde axonal transport after dorsal column lesion. J. Gene Med..

[52] Berry M (2001). Sustained effects of gene-activated matrices after CNS injury. Mol. Cell. Neurosci..

[53] Geller HM, Fawcett JW (2002). Building a bridge: engineering spinal cord repair. Exp. Neurol..

[54] Stokols S, Tuszynski MH (2004). The fabrication and characterization of linearly oriented nerve guidance scaffolds for spinal cord injury. Biomaterials.

[55] Houweling DA, Bar PR, Gispen WH, Joosten EA (1998). Spinal cord injury: Bridging the lesion and the role of neurotrophic factors in repair. Prog. Brain Res..

[56] King VR, Phillips JB, Hunt-Grubbe H, Brown R, Priestley JV (2006). Characterization of non-neuronal elements within fibronectin mats implanted into the damaged adult rat spinal cord. Biomaterials.

[57] King VR, Henseler M, Brown RA, Priestley JV (2003). Mats made from fibronectin support oriented growth of axons in the damaged spinal cord of the adult rat. Exp. Neurol..

[58] Bakshi A (2004). Mechanically engineered hydrogel scaffolds for axonal growth and angiogenesis after transplantation in spinal cord injury. J. Neurosurg. Spine.

[59] Piantino J, Burdick JA, Goldberg D, Langer R, Benowitz LI (2006). An injectable, biodegradable hydrogel for trophic factor delivery enhances axonal rewiring and improves performance after spinal cord injury. Exp. Neurol..

[60] Tate CC, Garcia AJ, LaPlaca MC (2007). Plasma fibronectin is neuroprotective following traumatic brain injury. Exp. Neurol..

[61] Sakai T (2001). Plasma fibronectin supports neuronal survival and reduces brain injury following transient focal cerebral ischemia but is not essential for skin-wound healing and hemostasis. Nat. Med..

[62] Letourneau PC, Madsen AM, Palm SL, Furcht LT (1988). Immunoreactivity for laminin in the developing ventral longitudinal pathway of the brain. Dev. Biol..

[63] Venstrom KA, Reichardt LF (1993). Extracellular matrix. 2: Role of extracellular matrix molecules and their receptors in the nervous system. FASEB J..

[64] Liesi P (1985). Laminin-immunoreactive glia distinguish regenerative adult CNS systems from non-regenerative ones. EMBO J..

[65] Frisen J (1995). Spinal axons in central nervous system scar tissue are closely related to laminin-immunoreactive astrocytes. Neuroscience.

[66] Matsui F, Oohira A (2004). Proteoglycans and injury of the central nervous system. Congenit Anom (Kyoto).

[67] Akhurst RJ (2006). A sweet link between TGFbeta and vascular disease?. Nat. Genet..

[68] Yamaguchi Y, Mann DM, Ruoslahti E (1990). Negative regulation of transforming growth factor-beta by the proteoglycan decorin. Nature.

[69] Renckens R (2005). The role of plasminogen activator inhibitor type 1 in the inflammatory response to local tissue injury. J. Thromb Haemost..

[70] Leon S, Yin Y, Nguyen J, Irwin N, Benowitz LI (2000). Lens injury stimulates axon regeneration in the mature rat optic nerve. J. Neurosci..

[71] Yin Y (2003). Macrophage-derived factors stimulate optic nerve regeneration. J. Neurosci..

[72] Yin Y (2006). Oncomodulin is a macrophage-derived signal for axon regeneration in retinal ganglion cells. Nat. Neurosci..

[73] Yin Y (2009). Oncomodulin links inflammation to optic nerve regeneration. Proc. Natl. Acad. Sci. U. S. A..

[74] Alizadeh A, Dyck SM, Karimi-Abdolrezaee S (2019). Traumatic spinal cord injury: An overview of pathophysiology, models and acute injury mechanisms. Front. Neurol..

[75] Nikravesh N (2019). Physical structuring of injectable polymeric systems to controllably deliver nanosized extracellular vesicles. Adv. Healthc. Mater..

[76] Ahmed Z (2005). Disinhibition of neurotrophin-induced dorsal root ganglion cell neurite outgrowth on CNS myelin by siRNA-mediated knockdown of NgR, p75NTR and Rho-A. Mol. Cell. Neurosci..

[77] Zivraj KH (2010). Subcellular profiling reveals distinct and developmentally regulated repertoire of growth cone mRNAs. J. Neurosci..

[78] Thompson A, Berry M, Logan A, Ahmed Z (2019). Activation of the BMP4/Smad1 pathway promotes retinal ganglion cell survival and axon regeneration. Invest. Ophthalmol. Vis. Sci..

[79] Read ML (2009). Profiling RNA interference (RNAi)-mediated toxicity in neural cultures for effective short interfering RNA design. J. Gene Med..

[80] Radtke C, Kocsis JD, Baumgartner W, Vogt PM (2017). Fluoro-Ruby as a reliable marker for regenerating fiber tracts. Innov. Surg. Sci..

[81] Ahmed Z (2005). Matrix metalloproteases: degradation of the inhibitory environment of the transected optic nerve and the scar by regenerating axons. Mol. Cell. Neurosci..

[82] Hata K (2006). RGMa inhibition promotes axonal growth and recovery after spinal cord injury. J Cell Biol.

[83] Hains BC, Saab CY, Lo AC, Waxman SG (2004). Sodium channel blockade with phenytoin protects spinal cord axons, enhances axonal conduction, and improves functional motor recovery after contusion SCI. Exp. Neurol..

[84] Lo AC, Saab CY, Black JA, Waxman SG (2003). Phenytoin protects spinal cord axons and preserves axonal conduction and neurological function in a model of neuroinflammation in vivo. J. Neurophysiol..

