# Supplementary material for: Thermosensitive collagen/fibrinogen gels loaded with decorin suppress lesion site cavitation and promote functional recovery after spinal cord injury
Source: Sci Rep. 2021 Sep 13;11:18124. doi: 10.1038/s41598-021-97604-w (PMC8438067; doi:10.1038/s41598-021-97604-w)
Supplement: Supplementary file 1 — Supplementary Figures. [file 41598_2021_97604_MOESM1_ESM.pdf]

Supplementary Information

Thermosensitive collagen/fibrinogen gels loaded with decorin suppress lesion site cavitation and promote functional recovery after spinal cord injury

Jacob Matthews[1], Sarina Surey[1], Liam M Grover[2], Ann Logan[3] & Zubair Ahmed[1,4*]

[1]Neuroscience and Ophthalmology, Institute of Inflammation and Ageing, University of Birmingham, Edgbaston, Birmingham, B15 2TT, UK

[2]School of Chemical Engineering, University of Birmingham, Edgbaston, Birmingham, B15 2TT, UK

[3]Biomedical Sciences, Warwick Medical School, University of Warwick, Coventry, CV4 7AL, UK.

4Centre for Trauma Sciences Research, University of Birmingham, Edgbaston, Birmingham, B15 2TT.

***Correspondence:** z.ahmed.1@bham.ac.uk; ORCID ID: 0000-0001-6267-6442

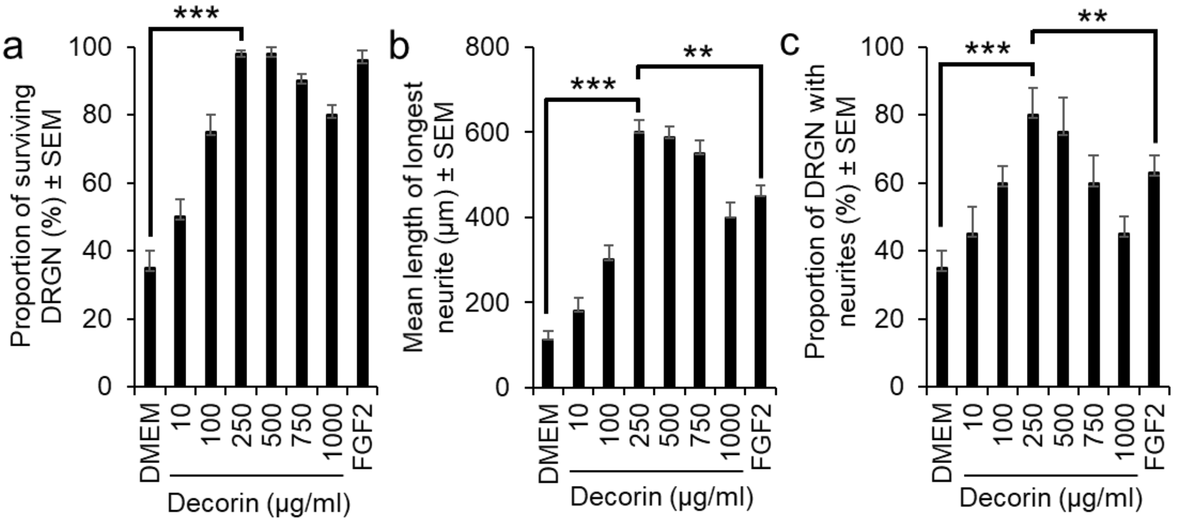

Supplementary Fig. S1. Dose response curve for Decorin in DRGN cultures *in vitro*. Decorin increased (a) DRNG survival, (b) DRGN neurite length and the (c) proportion of DRGN with neurites in a dose-dependent manner, with 250µg/ml being optimal. ** = P<0.001; *** = P<0.0001, one-way ANOVA with Dunnett's post hoc test. *n* = 3 wells/condition, 3 independent repeats, total *n* = 9 wells/condition.

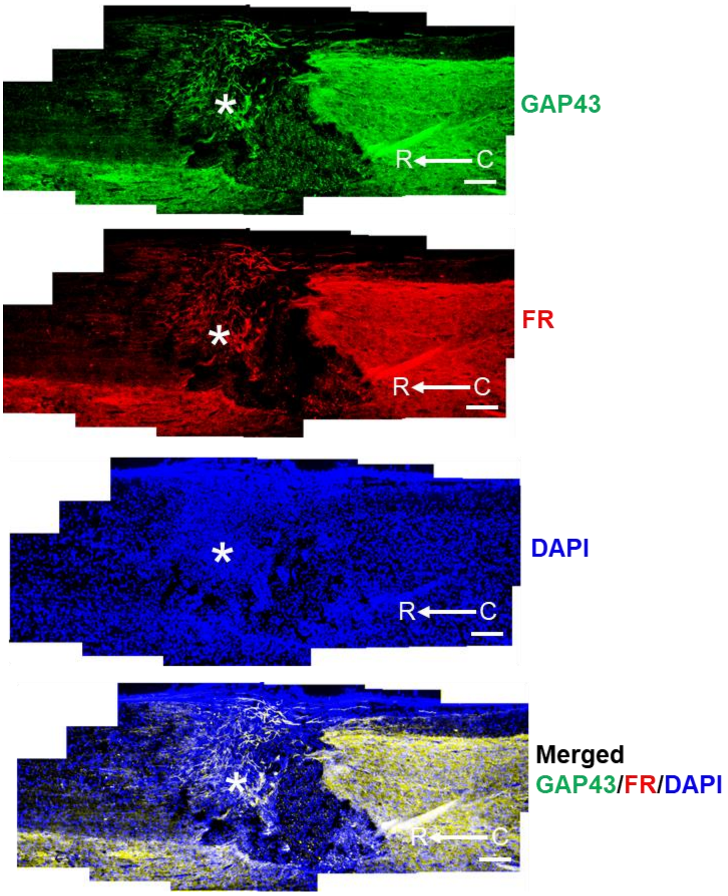

Supplementary Fig. S2. GAP43 immunostaining correlates with FR tracing of axons. GAP43 immunohistochemistry (green), FR traced axons (red), DAPI stained nuclei and merged images to show overlap of GAP43 with FR in the same section after treatment with Gel+Decorin. Scale bar = 200 µm. C to R = caudal to rostral direction of axon growth. * = lesion site.

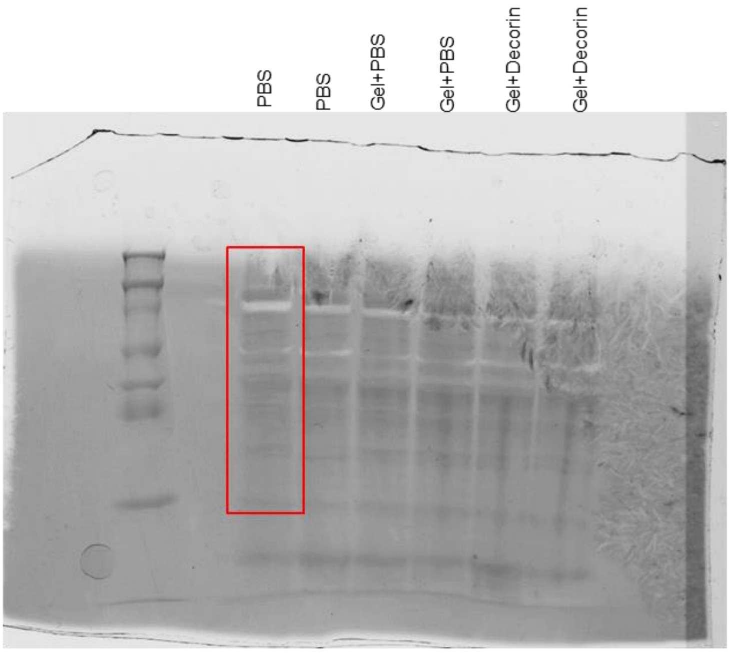

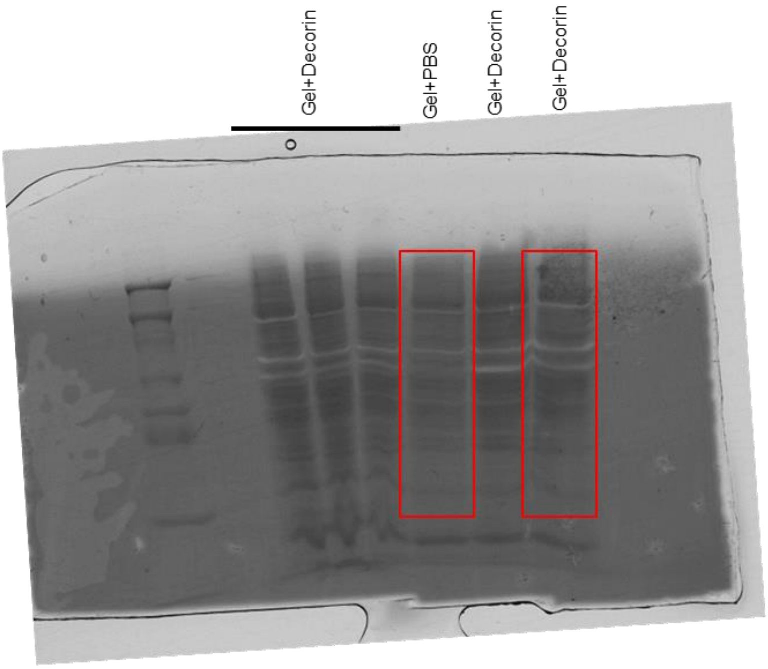

Supplementary Fig. S3. Full length zymograms from Figure 4a. Red boxes denote cropped areas in Figure 4a.

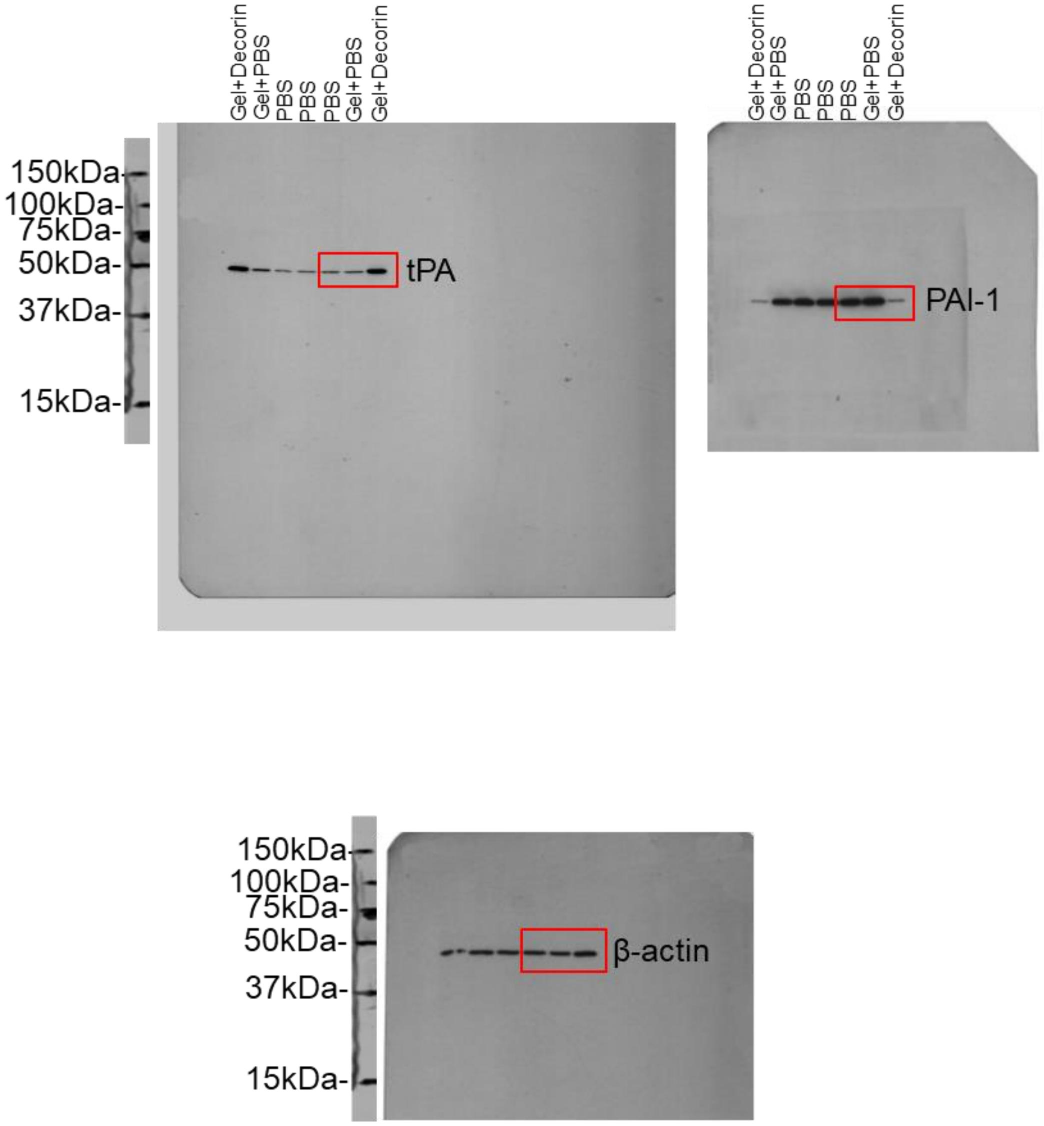

Supplementary Fig. S4. Full length blots from Figure 4d. Red box denotes cropped areas in Figure 4d.
